# Identification of anti-horn fly vaccine antigen candidates using a reverse vaccinology approach

**DOI:** 10.1186/s13071-021-04938-5

**Published:** 2021-09-03

**Authors:** Luísa N. Domingues, Kylie G. Bendele, Lénaïg Halos, Yovany Moreno, Christian Epe, Monica Figueiredo, Martin Liebstein, Felix D. Guerrero

**Affiliations:** 1 USDA-ARS Knipling-Bushland U. S. Livestock Insects Research Lab, 2700 Fredericksburg Road, Kerrville, TX USA; 2 Texas A&M University, Department of Entomology, 2475 TAMU, College Station, TX USA; 3 Boehringer Ingelheim Animal Health, 29 Avenue Tony Garnier, 69007 Lyon, France; 4 Bill and Melinda Gates Foundation, Seattle, WA USA; 5 Boehringer Ingelheim Animal Health, Pharmaceutical Discovery and Research, 3239 Satellite Blvd. Bldg. 600, Duluth, GA USA; 6 Boehringer Ingelheim Animal Health Missouri Research Center, 6498 Jade Rd, Fulton, MO USA

**Keywords:** *Haematobia irritans irritans*, Parasites, Biting flies, Cattle, Vaccines, Reverse vaccinology, In silico vaccine discovery, Peritrophic matrix, Peritrophins

## Abstract

**Background:**

The horn fly, *Haematobia irritans irritans*, causes significant production losses to the cattle industry. Horn fly control relies on insecticides; however, alternative control methods such as vaccines are needed due to the fly's capacity to quickly develop resistance to insecticides, and the pressure for eco-friendly options.

**Methods:**

We used a reverse vaccinology approach comprising three vaccine prediction and 11 annotation tools to evaluate and rank 79,542 translated open reading frames (ORFs) from the horn fly's transcriptome, and selected 10 transcript ORFs as vaccine candidates for expression in *Pichia pastoris*. The expression of the 10 selected transcripts and the proteins that they encoded were investigated in adult flies by reverse transcription polymerase chain reaction (RT-PCR) and mass spectrometry, respectively. Then, we evaluated the immunogenicity of a vaccine candidate in an immunization trial and the antigen’s effects on horn fly mortality and fecundity in an in vitro feeding assay.

**Results:**

Six of the ten vaccine candidate antigens were successfully expressed in *P. pastoris*. RT-PCR confirmed the expression of all six ORFs in adult fly RNA. One of the vaccine candidate antigens, BI-HS009, was expressed in sufficient quantity for immunogenicity and efficacy trials. The IgG titers of animals vaccinated with BI-HS009 plus adjuvant were significantly higher than those of animals vaccinated with buffer plus adjuvant only from days 42 to 112, with a peak on day 56. Progeny of horn flies feeding upon blood from animals vaccinated with BI-HS009 plus adjuvant collected on day 56 had 63% lower pupariation rate and 57% lower adult emergence than the control group (ANOVA: *F*_(1, 6)_ = 8.221, *P* = 0.028 and *F*_(1, 6)_ = 8.299, *P* = 0.028, respectively).

**Conclusions:**

The reverse vaccinology approach streamlined the discovery process by prioritizing possible vaccine antigen candidates. Through a thoughtful process of selection and in vivo and in vitro evaluations, we were able to identify a promising antigen for an anti-horn fly vaccine.

**Graphical abstract:**

**Figure:**
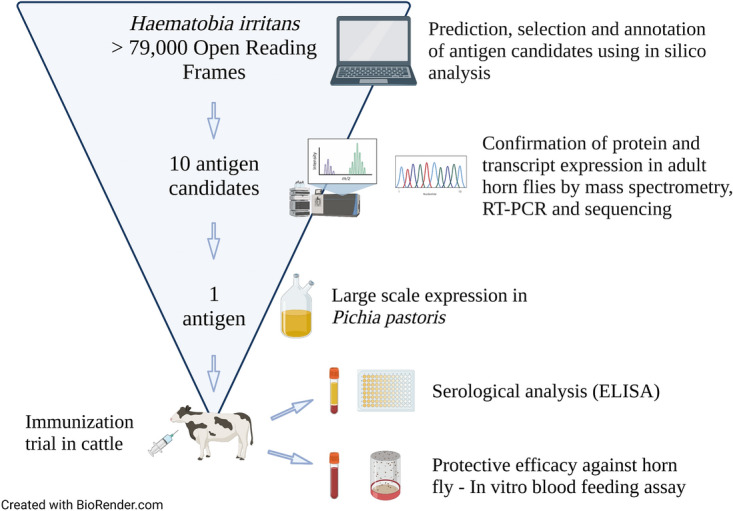

**Supplementary Information:**

The online version contains supplementary material available at 10.1186/s13071-021-04938-5.

## Background

The horn fly, *Haematobia irritans irritans* (Diptera: Muscidae) (Linnaeus, 1758), is present in central and southern Europe, Asia Minor, North Africa, and the Americas [1]. Large populations of this fly can cause significant blood loss and annoyance in livestock, reducing milk production, weaning weight, and weight gain, resulting in economic losses estimated at $876 million (~ $1.7 billion when adjusted for inflation using the Bureau of Labor Statistics calculator [2]) and $2.56 billion per year in the USA [3] and Brazil [4], respectively.

The intensive use of insecticides combined with the horn fly’s peculiar biology (high biotic potential, short life cycle, large number of generations per year, and close association with its host) has contributed to the quick selection of populations resistant to most of the products commercially available in the USA, including organochlorines (DDT), organophosphates, pyrethroids, and cyclodienes (endosulfan) [1, 5, 6]. Vaccines could be a valuable option for horn fly control. Their long-lasting effects reduce the need for insecticide treatments, they do not leave residues in animal-derived products or the environment, are highly specific with no side effects on non-target species, and have a low probability of selecting resistant populations [7, 8].

Reverse vaccinology is a genomic-based approach to vaccine development that uses computational and bioinformatic analyses of a pathogen’s genome, transcriptome, or proteome to predict antigens that are most likely to be successful vaccines [9]. This approach does not require pathogen cultivation like the traditional “isolate-inactivate-inject” principle and considers most antigens independent of their abundance and immunogenicity during infection, potentially being a faster and more economic method [9, 10]. Reverse vaccinology has been successfully used for the development of vaccines against viruses [11] and the prokaryote pathogen *Neisseria meningitidis* serogroup B [12].

No anti-horn fly vaccines are available commercially, and there is limited research published on this topic. A vaccine containing 1 mg of crude antigen extracted from horn fly intestine plus Freund’s incomplete and *Lactobacillus casei* adjuvants affected oviposition but not fly survival [13]. Flies that fed upon animals immunized with recombinant thrombostasin, an anti-thrombin peptide found in horn fly saliva, took smaller blood meals and delayed ovarian development compared to flies fed on unvaccinated cattle [14, 15]. Experimental vaccination with recombinant hematobin, a salivary protein, increased the cattle’s anti-hematobin IgG response and reduced fly loads by about 30% compared with the control group [16].

Considering the economic losses to the cattle industry caused by *H. irritans*, and the fly's resistance to current chemical control methods, novel technologies are needed to help control this pest. Molecular databases such as the horn fly genome [17] and transcriptomes [18–20] provide new tools and directions for the development of novel control technologies. The aim of this study was to use a reverse vaccinology approach to predict and rank anti-horn fly vaccine antigen candidates and evaluate their utility and efficacy as active ingredients in anti-horn fly vaccine formulations.

## Methods

### In silico selection of antigen candidates

**Fig. 1:**
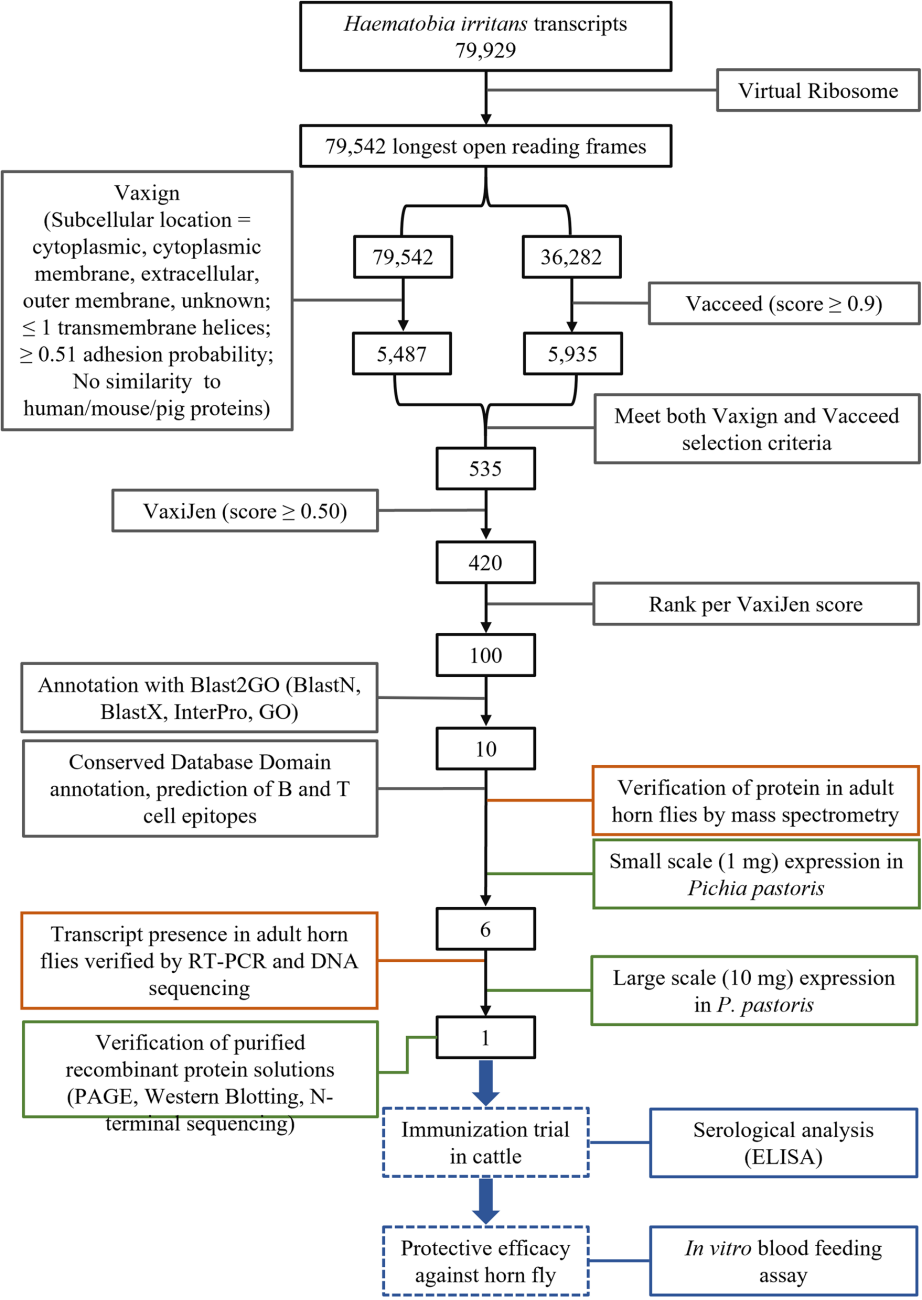
Schematic overview of the study design including in silico reverse vaccinology analysis steps for the selection of *Haematobia irritans* vaccine antigen candidates, amplification, and in vivo and in vitro evaluations

A full description of all tools used for the in silico analysis and the overall strategy used for antigen selection are shown in Table 1 and Fig. 1, respectively. A *H. irritans* transcriptome dataset with 79,929 sequences was used as the input for the in silico predictions (Additional file 1: Dataset S1). Sampling and RNA purification were performed as published previously [17, 19] and included assembled transcripts acquired from both male and female adult flies (assembled TSA Accession No. GGLM01000000), eggs (Accession No. SRX000777), and larvae (Accession No. SRX000776), as well as pupae, testes, ovaries, Malpighian tubules, forelegs, and adult female gut and salivary gland. Initially, the longest open reading frame (ORFs) of each transcript was determined using the Virtual Ribosome online tool [21] (Fig. 1; Table 1) set at default parameters, except as follows: reading frame—all (six reading frames), ORF finder—Start codon: Strict, stop codons—terminate.

**Table 1:** Tools used for the in silico analyses of *Haematobia irritans* transcripts and translated ORFs

Tool	Description	Website
Virtual Ribosome	Comprehensive tool for translating DNA sequences to the corresponding peptide sequences [21]	http://www.cbs.dtu.dk/services/VirtualRibosome/
Vaxign	Vaccine target prediction and analysis system based on the principle of reverse vaccinology [22–24]	http://www.violinet.org/vaxign/index.php
PSORTb	Program for bacterial protein subcellular localization prediction [29]	http://www.psort.org/psortb/
TMHMM	Prediction of transmembrane helices in proteins [30, 31]	https://services.healthtech.dtu.dk/service.php?TMHMM-2.0
SPAAN	Prediction of adhesins and adhesin-like proteins using neural networks [32]	-
BLAST	NCBI sequence similarity alignment and analysis program [33, 34]	https://blast.ncbi.nlm.nih.gov/Blast.cgi
IEDB	Immune Epitope Database and Analysis Resource [35]	http://www.iedb.org/
Vacceed	High-throughput in silico vaccine candidate discovery pipeline for eukaryotic pathogens based on reverse vaccinology [25, 26]	-
WoLF PSORT	Protein subcellular localization prediction [36]	https://wolfpsort.hgc.jp/
SignalP 4.1	Predicts presence and location of signal peptide cleavage sites [37]	http://www.cbs.dtu.dk/services/SignalP/
TargetP 1.1	Predicts the subcellular location of eukaryotic proteins [38]	http://www.cbs.dtu.dk/services/TargetP/
TMHMM	Prediction of transmembrane helices in proteins [30]	TMHMM—2.0—Services—DTU Health Tech
MHC I-binding	Peptide binding to MHC class I molecules [39]	http://tools.immuneepitope.org/mhci/
MHC II-binding	Peptide binding to MHC class II molecules [40]	http://tools.immuneepitope.org/mhcii/
VaxiJen	Server for alignment-independent prediction of protective antigens. It allows antigen classification solely based on the physicochemical properties of proteins without recourse to sequence alignment [27, 28]	www.ddg-pharmfac.net/vaxijen/VaxiJen/VaxiJen.html
Blast2GOPro	Bioinformatics platform for the functional analysis of genomic datasets [44]	www.blast2go.com
BLASTN	Finds regions of similarity between nucleotide sequences using a nucleotide query [33, 34]	https://blast.ncbi.nlm.nih.gov/Blast.cgi?PROGRAM=blastn&PAGE_TYPE=BlastSearch&BLAST_SPEC=&LINK_LOC=blasttab&LAST_PAGE=blastn
BLASTX	Finds regions of similarity between protein sequences using a translated nucleotide query translated in all six reading frames [33, 34]	https://blast.ncbi.nlm.nih.gov/Blast.cgi?PROGRAM=blastx&PAGE_TYPE=BlastSearch&BLAST_SPEC=&LINK_LOC=blasttab&LAST_PAGE=blastx
InterPro	Provides functional analysis of protein sequences by classifying them into families and predicting the presence of domains and important sites. It uses predictive models known as signatures provided by several different databases [41]	https://www.ebi.ac.uk/interpro/
Gene Ontology (GO)	Defines concepts/classes used to describe gene function, and relationships between these concepts. It classifies functions along three aspects: molecular function, cellular component, biological process [42, 43]	http://geneontology.org/
Conserved Domain Database	Protein annotation resource that consists of a collection of well-annotated multiple sequence alignment models for ancient domains and full-length proteins [45, 46]	https://www.ncbi.nlm.nih.gov/Structure/bwrpsb/bwrpsb.cgi
BepiPred 2.0	Predicts B-cell epitopes from a protein sequence, using a random forest algorithm trained on epitopes and non-epitope amino acids determined from crystal structures. A sequential prediction smoothing is performed afterwards [47]	http://www.cbs.dtu.dk/services/BepiPred/
FBCPred	Predicts flexible length B-cell epitopes using subsequence kernel [48]	http://ailab.ist.psu.edu/bcpred/predict.html
BCPred	Predicts linear B-cell epitopes using string kernels [49]	http://ailab.ist.psu.edu/bcpred/predict.html
NetMHC 4.0 Server	Predicts peptide-MHC class I binding using artificial neural networks, and peptides are classified as having a strong or weak binder according to their ranking [50, 51]	http://www.cbs.dtu.dk/services/NetMHC/
IEDB-MHC I Binding Predictions	Predicts peptide-MHC class I binding using a combination of artificial neural network, stabilized matrix method, and scoring matrices derived from combinatorial peptide libraries. Predictions were made on 06 February 2018 [35, 50, 52–55]	http://tools.iedb.org/mhci/

Subsequently, the translated ORFs (Additional File 2: Dataset S2) were evaluated using Vaxign [22–24], Vacceed [25, 26], and VaxiJen [27, 28] (Fig. 1; Table 1), vaccine target prediction tools based on the principles of reverse vaccinology, i.e. the use of computational methods and tools to analyze the genetic information of a pathogen and predict antigens that are most likely to be vaccine candidates [9].

Vaxign includes a pipeline of software programs (PSORTb [29], TMHMM [30, 31], SPAAN [32], BLAST [33, 34], IEDB [35]) and predicts possible vaccine targets based on antigen subcellular location, adhesion, epitope binding to MHC class I and class II, and little if any sequence similarity to human, mouse, and/or pig proteins (Table 1). Peptide sequences that met the following criteria were chosen for further analysis: subcellular location—cytoplasmic, cytoplasmic membrane, extracellular, outer membrane, or unknown subcellular localization; ≤ 1 transmembrane helix, ≥ 0.51 adhesion probability; no similarity to human or mouse or pig proteins (Fig. 1).

In parallel to the Vaxign analysis, the translated ORFs were analyzed using Vacceed (Table 1; Fig. 1). To comply with the program requirements, only sequences with ≥ 100 amino acids were analyzed (*n* = 36,282) (Fig. 1). The Vacceed pipeline includes six programs (WoLF PSORT [36], SignalP 4.1 [37], TargetP 1.1 [38], TMHMM [30], MHC I Binding Predictor v2.5 [39], and MHC II Binding Predictors v2.5.1 [40]) that predict characteristics relevant to subcellular location, transmembrane helices, and ability to bind to MHC class I and II molecules (Table 1).

Subsequently, we merged the Vaxign and Vacceed results, and sequences that met the criteria for both programs were analyzed using VaxiJen, a server for alignment-independent prediction of protective antigens (Table 1; Fig. 1). VaxiJen classifies proteins using a score system, and proteins with a score of ≥ 0.51 are considered probable antigens (Fig. 1).

To gather more information about the predicted antigens, we analyzed the 100 highest ranked ORFs, based upon the VaxiJen score, using BLASTN [33, 34], BLASTX [33, 34], InterPro [41], and Gene Ontology (GO) [42, 43] annotation on BLAST2GOPro [44] (Table 1; Fig. 1). The following databases were used for the BLAST searches: for BLASTN, we used Transcriptome Shotgun Assembly (TSA) (an archive of computationally assembled mRNA sequence primary data such as EST and raw sequence reads) and Expressed Sequence Tags (EST) (in GenBank + EMBL + DDBJ + sequences from EST divisions of NCBI); for BLASTX we used Non-redundant (NR) (all non-redundant GenBank CDS translations + PDB + Swiss-Prot + PIR + PRF, excluding environmental samples from WGS projects). All analyses were performed using an e-value of *e*^−3^, a conservative value chosen to ensure retention and detection of distant orthologs. InterPro and GO annotations were performed using the default parameters on BLAST2GO.

Finally, we carefully evaluated the annotations of those 100 highest ranked ORFs and selected 10 candidates for in vitro expression.

### In silico characterization of selected antigens

The 10 antigens selected for in vitro expression were further annotated using the Conserved Domain Database (CDD) [45, 46] and B- and T-cell epitope prediction tools (Table 1; Fig. 1). CDD content includes NCBI-curated domains as well as Pfam, SMART, COG, PRK, and TIGRFAMS domains. The CDD database was searched using the default parameters, except for search against database = CDD – 50369 PSSMS, and expect value threshold = 0.001.

The following online tools were used for B-cell epitope prediction: BepiPred 2.0 [47], FBCPred [48], and BCPred [49] (Table 1; Fig. 1). For BepiPred 2.0, epitopes classified as exposed and with an epitope probability ≥ 0.5 were considered strong epitopes, while those classified as hidden and with an epitope probability ≥ 0.5 were classified as weak. Parameters used for FBCPred and BCPred analyses were 14- and 20-epitope lengths, respectively, and 75% specificity.

NetMHC 4.0 Server [50, 51] and IEDB-MHC I Binding Predictions [35, 50, 52–55] were used for T-cell epitope predictions (Table 1; Fig. 1). The parameters used for NetMHC 4.0 were 9mer peptides, bovine alleles (BoLA-D18.4, BoLA-HD6, BoLA-JSP.1, BoLA-T2a, BoLA-T2b, Bo-LA-T2C), and a threshold of ≤ 0.5 and > 0.5% but ≤ 2% rank for strong and weak binders, respectively. The parameters used for the IEDB-MHC I binding predictions included: prediction method—IEDB-recommended; MHC source species —cow; MHC alleles—all 87 bovine alleles; length—nine; sort peptides by—position in sequence; show—all predictions; cutoff—percentile rank ≤ 1%.

### Confirmation of protein expression in adult flies

To determine whether the 10 vaccine candidate antigens selected for in vitro expression were present in the horn fly, proteins were extracted from adult flies and analyzed by mass spectrometry. The adult flies used in the study were from the reference laboratory strain that has been kept in colony feeding upon citrated bovine blood at the United States Department of Agriculture (USDA)–Agricultural Research Services (ARS) Knipling-Bushland US Livestock Insects Research Laboratory (KBUSLIRL) for over 50 years [56].

Aqueous- and urea-extractible proteins were extracted using the ReadyPrep Sequential Extraction Kit (Bio-Rad) following the manufacturer’s recommendation, with a few modifications. Briefly, two samples of 100 adult flies of mixed sex from unfed and citrated blood-fed populations were separately ground on ice with a disposable pestle for 30 s followed by 10 s of centrifugation at 16,100×*g*, repeating this twice. Aqueous-soluble proteins were extracted by adding Reagent 1 (40 mM Tris) (ReadyPrep Sequential Extraction Kit) (Bio-Rad, Hercules, CA, USA) and FOCUS ProteaseArrest (G-Bioscience, St. Louis, MO, USA) to the samples and grinding with a Polytron homogenizer (PCU-2-110) (Brinkmann Instruments, Westbury, NY, USA) for 10 s, and returned to ice for 30 s, repeating this twice. Ribonuclease A from bovine pancreas (Sigma Aldrich, St. Louis, MO, USA) was added, and the samples were incubated at room temperature for 45 min on an orbital shaker at 225 rpm. Subsequently, samples were centrifuged for 30 min at 16,100×*g* at room temperature, and the supernatant was removed and saved as the Reagent 1 fraction, containing the aqueous-solubilized proteins. The pellet was washed three times using Reagent 1 plus FOCUS ProteaseArrest (G-Bioscience) and centrifuged for 30 min at 16,100×*g* at room temperature. The supernatant was discarded after each centrifugation, and after the final wash, the pellet was resuspended with Reagent 2 (8 M urea, 4% w/v CHAPS, 40 mM Tris, 0.2% w/v BioLyte 3/10 ampholyte) + tributylphosphine (TBP) (ReadyPrep Sequential Extraction Kit) (Bio-Rad), followed by incubation at room temperature for 1 h with shaking at 225 rpm and centrifugation as previously described. This supernatant was recovered as the Reagent 2 fraction, containing the 8 M urea-solubilized proteins.

Reagent 1 and 2 fractions were analyzed by SDS-PAGE under denaturing conditions using 1X Tris/Glycine/SDS buffer (Bio-Rad) and 4% acrylamide (AA)/bis-acrylamide (bis) stacking gel and 12% AA/bis resolving gel. Gels were stained using Coomassie Brilliant Blue R-250 (Bio-Rad). The gel was manually cut, and pieces containing proteins of 10–250 kDa were used in the mass spectrometry analysis.

Mass spectrometry (MS) was performed at the Department of Chemistry of the University of Georgia. Briefly, the gel pieces were digested with trypsin, and a list of precursor ions was generated from the provided protein sequences. The mass-to-charge values (m/z) of theoretical tryptic peptides of these proteins with up to one missed cleavage and multiple possible charges were calculated to make up a target ion list. Only the peptides with molecular weights between 0.5 and 3 kDa were included in the target ion list. Using this target ion list, a customized MS acquisition method was generated for the LC/MS runs. During the LC/MS run, a survey MS scan measured all ions from m/z 350–1800 and generated a peak list. The computer matched the peak list of the survey scan in the target ion list. The most intense eight peaks were analyzed by MS/MS, and if no ions from the target ion list were observed, the program picked eight ions that had the highest chance to produce good MS/MS spectra. Once those ions were analyzed by MS/MS, the program found the next possible candidates by doing another MS survey scan and looking at the peaks that were eluting, repeating the cycle described above. The LC/MS data were searched against the NCBI protein database (4837 horn fly sequences as of January 8, 2018) and the translated ORFs (Additional File 2: Dataset S2) used in the in silico analysis using Mascot (Matrix Sciences, Boston, MA, USA) combined with Proteome Discoverer (Thermo Fisher Scientific, Carlsbad, CA, USA).

### Confirmation of transcript expression in adult flies

To check if the mRNA transcripts of the vaccine antigens that were expressed in vitro were expressed in field populations of the horn fly (Fig. 1), we performed RT-PCR and DNA sequencing of adult flies from various populations sampled over the years and stored at −80 °C for preservation of nucleic acids. All fly samples were collected using sweep nets, transferred into a plastic collection tube, and set into dry ice or flash-frozen in liquid nitrogen.

Wild flies were collected at the Louisiana State University (LSU) AgResearch Saint Gabriel research farm in the state of Louisiana in 2006. Total RNA was extracted from 50 female flies using the ToTALLY RNA kit (Ambion Inc., Austin, TX, USA), followed by polyA RNA isolation using the MicroPoly(A)Purist Kit (Ambion Inc.) and cDNA synthesized using the SMART RACE cDNA Amplification kit (Clontech, Mountain View, CA, USA) following the manufacturers' recommendations.

Flies from Rosepine (wild flies collected at the Rosepine LSU AgResearch farm in the state of Louisiana in 1998) and Super Resistant (a since discontinued laboratory strain fed upon a stanchioned steer at KBUSLIRL from 1996 to 2006, sampled in May 1998) were also used in the study. For these samples, total RNA was extracted from a pool of adult males and females (10 each) using TRIzol reagent/chloroform/isopropanol. Briefly, tubes containing the flies were immersed in liquid nitrogen, transferred into dry ice, and pulverized using a mini-pestle. One ml of TRIzol reagent (Ambion by Life Technologies, Carlsbad, CA, USA) was added per 100 mg of tissue and mixed well with a mini-pestle, followed by incubation for 5 min at room temperature. Then, 0.2 ml of chloroform (Eastman Kodak Co., Rochester, NY, USA) per 1 ml of TRIzol reagent was added, and the tubes were vortexed for 30 s and incubated for 3 min at room temperature. Subsequently, tubes were centrifuged for 15 min at 12,000×*g* and 4 °C. The upper aqueous phase was recovered, and 0.5 ml of isopropanol (Sigma Aldrich) was added per 1 ml of TRIzol reagent. The sample was vortexed and incubated for 30 min at room temperature, followed by centrifugation for 10 min at 12,000×*g* and 4 °C. The supernatant was discarded, and the pellet was rinsed with 1 ml of 75% ethanol (Pharmco, Brookfield, CT, USA) per 1 ml of TRIzol reagent by vortexing. Samples were centrifuged for 5 min at 7500×*g* and 4 °C twice, and the supernatant discarded after each centrifugation. The remaining pellet was air-dried for 5 min at room temperature and resuspended in 50 µl nuclease-free water (Ambion Inc.). Subsequently, the total RNA was DNAse-treated using the RNase-Free DNase Set (Qiagen, Hilden, Germany) and the RNeasy Mini Kit (Qiagen) following the manufacturer’s recommendations. cDNA was synthesized using the RETROscript Kit (Invitrogen by Thermo Fisher Scientific, Vilnius, Lithuania) following the manufacturer’s recommendations.

Amplifications were performed on 25 µl PCR reactions with 1 µl of cDNA, 1X Q5 Reaction Buffer (New England Biolabs), 200 µM of dNTPs (Applied Biosystems, Foster City, CA, USA), 0.5 µM each of primers (Additional file 3: Table S1), 0.02 U/µl of Q5 Hot Start High-Fidelity DNA Polymerase (New England Biolabs), and 1X Q5 High GC Enhancer (New England Biolabs). Amplification was carried out using a DNA Engine preheated to 98 °C and programmed to 30 s at 98 °C, followed by either 30 or 35 cycles of denaturation at 98 °C for 10 s, annealing at different temperatures depending on the primer pair (Additional file 3: Table S1) for 30 s, and extension at 72 °C for 30 s. A final extension of 72 °C for 2 min was also included. The PCR product was purified by agarose gel electrophoresis, and the single products were extracted and purified using the QIAquick Gel Extraction Kit (Qiagen Inc., Valencia, CA, USA) per the manufacturer’s protocols. DNA sequencing was performed by Retrogen Inc. (San Diego, CA, USA), sequencing both strands to ensure accurate results. Primers used for sequencing are listed in Additional file 3: Table S1. MacVector version 15.1.4 with Assembler (MacVector Inc., Cary, NC, USA) was used for sequence assembly and nucleotide alignments.

### Recombinant expression of selected candidates in *Pichia pastoris*

The proteins selected for in vitro expression were contracted to Creative BioMart (Shirley, NY, USA) for cloning and recombinant expression in *P. pastoris*. Briefly, the recombinant DNA was synthesized using codons optimized for *P. pastoris* expression. Clones producing the recombinant proteins were grown in BMGY (1% yeast extract, 2% peptone, 100 mM potassium phosphate pH 6.0, 1.34% yeast nitrogen base with ammonium sulfate without amino acids, 4 × 10^–5^% biotin, 1% glycerol) or BMMY (BMGY but substituting 0.5% methanol for the 1% glycerol) media for 96 h. Every 24 h, methanol was added to a final concentration of 1% to maintain induction. Cells were harvested by centrifugation 96 h post induction. Recombinant proteins were purified making use of the 6X-histidine tag supplied by the vector sequence and Ni^2+^-NTA resin with a binding buffer composed of phosphate-buffered saline (PBS) (pH 7.5) and 10% glycerol. Wash and elution buffers were composed of 0, 30, 50, 200, and 4000 mM imidazole. The final product was suspended in PBS (pH 7.5) and 50% glycerol and stored at −20 °C. A 1-mg quantity of each protein was targeted for the initial small-scale tests. Subsequently, a 10-mg large-scale expression was required for those used in the vaccine trial reported herein.

### Verification of purified recombinant protein

Production of recombinant proteins was verified by SDS-PAGE, Western blotting, and N-terminal sequencing. For the SDS-PAGE, the recombinant protein was resolved in NuPAGE 4–12% Bis–Tris gel 1 mm × 12 wells (Invitrogen by Thermo Fisher Scientific) with MES Running Buffer (Invitrogen by Thermo Fisher Scientific) under denaturing conditions. Gels were stained using Coomassie Brilliant Blue R-250 (Bio-Rad), and gel images were saved using Bio-Rad Gel Doc EZ Imager and Image Lab 3.0 Software (Bio-Rad).

For the Western blotting, after the SDS-PAGE, the recombinant protein was transferred to 0.45 µM nitrocellulose membranes (Novex by Life Technologies, Carlsbad, CA, USA), and detection was performed using the Western Breeze Chromogenic Western Blot Immunodetection Kit (Invitrogen by Thermo Fisher Scientific) and anti-his(C-term)-HRP antibody (Novex by Life Technologies) following the manufacturer’s recommendation.

Additionally, N-terminal sequencing was used to verify the expressed recombinant protein containing the correct amino acids. Briefly, the protein was resolved by SDS-PAGE as previously described, then transferred to Sequi-blot PVDF membranes (Bio-Rad) using a blotting buffer composed of 1X CAPS pH 11 (Sigma Aldrich) and 10% methanol (Sigma Aldrich) and stained with Coomassie Brilliant Blue R-250. The membrane was shipped to the Molecular Structure Facility, University of California, Davis (Davis, CA, USA), where the protein was sequenced with the Procise 494 system (2) (Applied Biosystems).

### Immunization trial

#### Study design

An immunization trial was performed at the Missouri Research Center of Boehringer Ingelheim (Fulton, MO, USA) to evaluate antigen immunogenicity and safety to cattle. Animals were housed on pastures and received water ad libitum as well as a grain supplement. Twelve castrated male Holstein calves, approximately 18 months old, were randomly distributed into two groups (BI-HS009 and control), as follows: on day 5, 12 animals were ranked in ascending alpha-numerical identification order (tag number). The first eight animals were assigned to four blocks with two animals per block. The animal with the lowest random number was assigned to BI-HS009, and the animal with the highest random number was assigned to the control group. The remaining four animals were assigned to BI-HS009, totaling eight and four animals in the BI-HS009 and control groups, respectively.

Animals from the BI-HS009 group were vaccinated subcutaneously with three doses of 114 µg/dose (4 ml dose) of BI-HS009 antigens in PBS (pH 7.5) plus 50% glycerol with adjuvant on days 0, 21, and 42. The control group received a formulation containing only buffer and adjuvant (4 ml dose) on the same days as the BI-HS009 group. The adjuvant used for all groups consisted of saponin, aluminum, and TS6 (Boehringer Ingelheim’s proprietary adjuvant). Since the total dose contained a relatively large volume of adjuvant, the 4 mL dose was split and administered in two injection sites, one on each side of the animal at each vaccination time point.

Blood was collected before vaccination on days 0, 21, and 42 as well as on days 56, 70, 84, 98, and 112 using sodium heparin tubes (Becton, Dickinson and Company, Franklin Lakes, NJ, USA) for whole blood samples, and serum separator tubes for serum samples (Becton, Dickinson and Company). Serum samples were centrifuged, separated into aliquots, and stored at −20 °C. Whole blood tubes were kept refrigerated at 4 °C. The serum and blood samples were shipped overnight to KBUSLIRL where they were subjected to ELISA or used for in vitro horn fly feeding, respectively.

All protocols for animal studies were reviewed and approved by Boehringer Ingelheim’s Institutional Animal Care and Use Committee (Animal Procurement Statement 19–62).

#### Serological analysis

The antibody response of each vaccinated animal was analyzed using an indirect ELISA. Briefly, the recombinant protein used in the immunization trial was diluted in BupH Carbonate-Bicarbonate Buffer (Thermo Fisher Scientific, Rockford, IL, USA) to a final concentration of 0.25 µg/ml. One hundred µl of diluted antigen was added to each well of a 96-well plate (Thermo Fisher Scientific), and the plate was incubated overnight at room temperature. The wells were emptied and blocked with 300 µl of Blocker BLOTTO in TBS (Thermo Fisher Scientific) for 1 h. Serum dilutions ranging from undiluted to 1:4000 were prepared in 1X TBS, 0.05% Tween 20, and 10% Blocker BLOTTO (Thermo Fisher Scientific) and added to each well and incubated for 1.5 h. The plate was then rinsed four times with 1X TBS Tween 20 Buffer (Thermo Fisher Scientific) to remove unbound serum components, and 100 µl of peroxidase-labeled rabbit anti-bovine IgG (H+L) (0.05 µg/ml) (KPL, Gaithersburg, MD, USA) was added to each well, followed by 1 h of incubation at room temperature. The plate was rinsed again four times with 1X TBS Tween 20 buffer, and 100 µl of TMB (3,3′,5,5′-tetramethylbenzidine) substrate solution (Thermo Fisher Scientific) added, followed by 20 min of incubation. Finally, the reaction was stopped by adding 100 µl Stop Solution (Thermo Fisher Scientific), and absorbance (OD450) was read using an ELX800 plate reader (BioTek Instruments, Winooski, VT, USA). Standards, samples, and blanks were run in triplicate. The antibody titers of the samples were expressed as antibody units determined relative to a standard curve [57]. Calculations were performed using Gen5 version 2.05 software (BioTek Instruments).

### Protective efficacy against horn fly

The protective efficacy of BI-HS009 was tested using an in vitro feeding assay performed at KBUSLIRL using 1-day-old, unfed adult horn flies from the KBUSLIRL reference susceptible colony [56]. Blood samples from day 21, 42, or 56 were used for the feeding trials, and samples collected from all animals within a group (BI-HS009 or control) were pooled for each day. The blood was kept at 4 °C throughout the study.

**Fig. 2:**
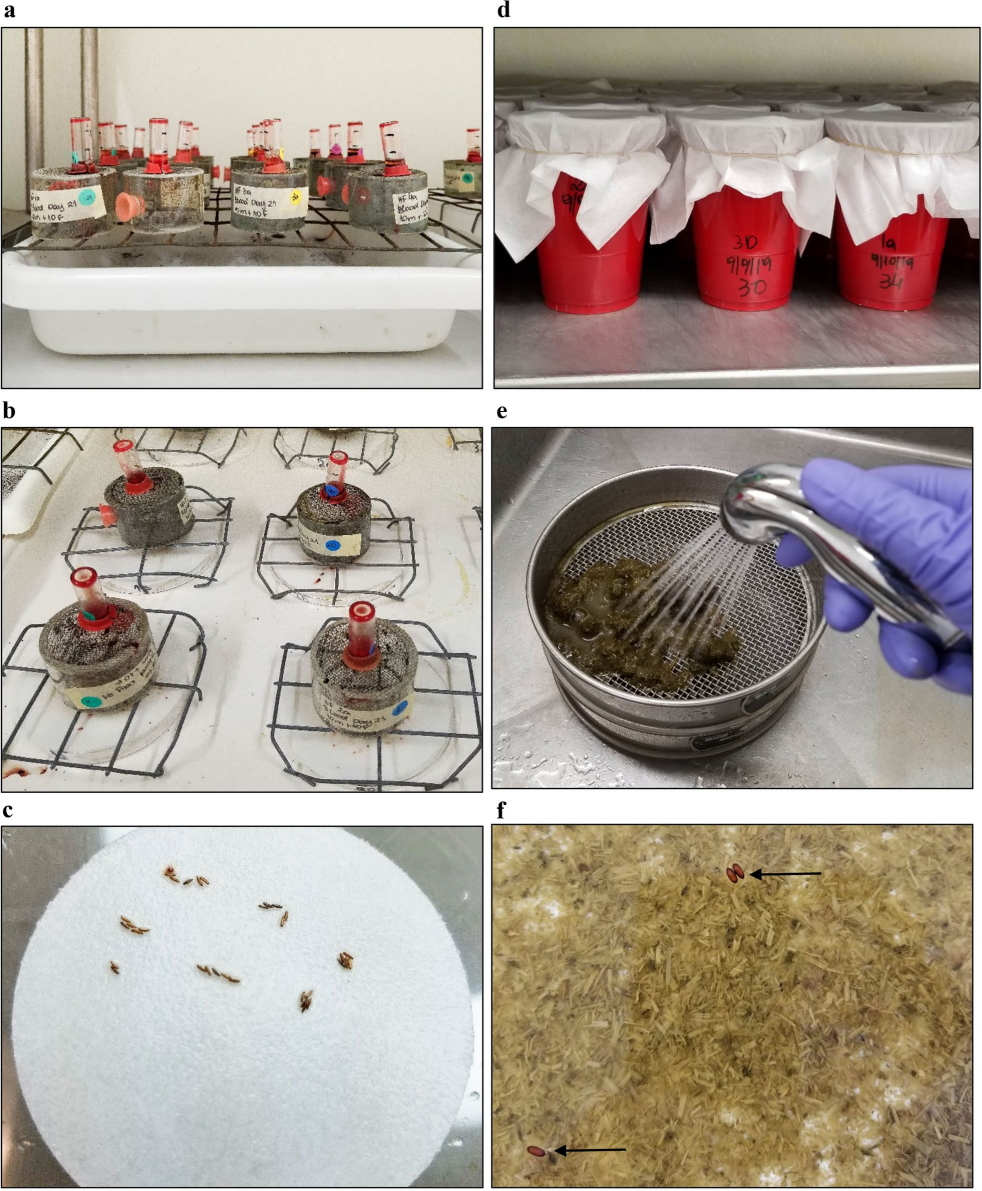
Horn fly in vitro feeding trial. **a** Adult flies in cages feeding for 10 days upon whole blood collected on days 21, 42, or 56 from animals vaccinated with BI-HS009 or buffer plus adjuvant only. (**b** Egg collection by allowing oviposition onto moistened filter pads. **c** Fly eggs on filter paper. **d** Larval feeding cups containing manure. **e** Pupae recovered by washing manure onto sieves of different sizes. **f** Pupae (indicated by arrows) recovered by flotation in a container filled with tap water

The flies were kept in acrylic screened cages (4.8 cm diameter, 4.5 cm height) with a 1.25-cm access hole closed with #5 cap plugs (Protective Closures Co., Buffalo, NY, USA) (Fig. 2a). Four cages with 20 flies each (10 males and 10 females) were used per group (BI-HS009 and control) for each blood collection date (day 21, 42, or 56).

Flies were fed twice a day: glass vials (12 × 35 mm, 0.5 dram) (Kimble, China) containing 0.5 ml of blood were placed on top of the cages at 9 am each day and then replaced at 3 pm with 1.5 ml of fresh blood (Fig. 2a, b). Dead flies were recovered from cages daily and the number recorded. On days 6, 7, and 8, eggs were collected by setting cages atop a Whatman filter paper 1 quantitative 90 mm diameter circle (Whatman International, Maidstone, England) placed onto a water-soaked all-natural cotton classic contour pad (Maxim Hygiene, New York) set in a 15 × 100-mm petri dish (Falcon, Durham, NC, USA). A wire rack was placed on top of the filter paper/cotton pad/petri dish configuration to elevate the cage above the wet materials, and the cage with flies was placed on top of the wire rack (Fig. 2b). Eggs were collected for 3 h, then counted with the aid of a magnifying glass (Fig. 2c) and placed in plastic cups (18 oz.) (Solo Cup Company, Lake Forest, IL, USA) containing 100 g of cow manure. The cups were covered with tissue paper (LC Industries, Durham, NC, USA) secured with rubber bands (size 54) (Skilcraft, Utica, NY, USA) and incubated at 29 °C (Fig. 2d). On days 13, 14, and 15, pupae were recovered from the manure with the aid of two sieves, number 7 (2.80 mm) and number 20 (850 µm) (W. S. Tyler, Mentor, OH, USA) (Fig. 2e, f) followed by flotation in a container filled with tap water and placed in Petri dishes (100 × 15 mm) (Falcon) lined with a Whatman filter paper 1 quantitative 90 mm diameter circle (Whatman International). The number of adults that emerged from the pupae were counted on days 17, 18, and 19.

The protective efficacy of the blood of vaccinated animals against horn fly was measured using the following parameters: number of dead fed adult flies (fly mortality), number of eggs laid by fed flies, number of progeny pupae, and number of newly emerged adults.

### Statistical analysis

The antibody response of each animal on each day measured by indirect ELISA was log transformed to account for outliers. The transformed ELISA results as well as the number of dead fed adult flies (fly mortality), number of eggs laid by fed flies, number of progeny pupae, and number of newly emerged adults of both groups were compared using two-way repeated-measures ANOVA followed by the Šídák’s multiple comparisons test. In addition, the treatment effects on egg to pupariation and puparia to adult development were analyzed using multiple logistic regression with pupae and emerging adults as the dependent variables. All the analyses were performed using GraphPad Prism version 8.2.1 for Windows (GraphPad Software, La Jolla California, USA), and differences were considered statistically significant when *P* < 0.05.

## Results

### In silico selection of antigen candidates

Virtual Ribosome ORF finding and translation of the 79,929-transcript dataset (Additional file 1: Dataset S1) resulted in 79,542 predicted ORFs (Fig. 1, Additional file 2: Dataset S2). A small number of transcripts (*n* = 387) did not produce an ORF because they did not have the methionine start codon (ATG), one of the selection criteria we chose for finding ORFs.

The Vaxign analysis of the 79,542 ORFs resulted in 5487 ORFs predicted as vaccine antigen candidates according to our selection criteria (Fig. 1, Additional file 4: Dataset S3). The Vacceed analysis produced a ranked output of the 36,282 candidates analyzed using this tool (Fig. 1, Additional file 4: Dataset S3). To further pare down these results into a workable dataset, all the ORFs with Vacceed scores above 0.9 (*n* = 5,935) that were also present in the 5487 candidates from the Vaxign analysis were pooled into a dataset of 535 ORFs (Fig. 1, Additional file 4: Dataset S3). These 535 ORFs were analyzed with VaxiJen, and 420 were considered probable candidates by this tool (VaxiJen score > 0.5) (Fig. 1, Additional file 4: Dataset S3). These 420 ORFs were ranked by VaxiJen analysis scores, and the 100 ORFs with the highest score were selected for manual annotation, described above (Fig. 1, Additional file 4: Dataset S3). We manually inspected all the acquired annotation of the top 100, looking at predicted protein function, localization within the cell, solubility, life stage, and organ of expression in dipterans (when available), lack of amino acid similarity to mammalian proteins, presence in Diptera databases, and other information (Additional file 4: Dataset S3), and selected 10 candidates for recombinant expression in *P. pastoris*. Our criteria for vaccine antigen candidate selection were developed from studies on cattle ticks and are described in Guerrero et al*. *[58].

The 10 vaccine candidates were designated as BI-HS001–BI-HS010, and their contig ID, transcript, and ORF sequences can be found in Additional file 5: Dataset S4. The Vacceed score of BI-HS001–BI-HS010 ranged from 0.930 (BI-HS005) to 0.999 (BI-HS001), and their ranking among all the 36,282 sequences analyzed with Vacceed ranged from 32 (BI-HS001) to 5459 (BI-HS005) (Table 2, Additional file 5: Dataset S4). VaxiJen scores of BI-HS001–BI-HS010 ranged from 0.8107 (BI-HS010) to 1.5791 (BI-HS001), while their rankings among the 535 sequences analyzed with VaxiJen ranged from 16 (BI-HS001) to 90 (BI-HS010) (Table 2, Additional file 5: Dataset S4).

**Table 2:** Summary for the 10 selected vaccine antigen candidates

Antigen ID	Vacceed score (rank)^a^	VaxiJen score (rank)^b^	MS confirmed^c^	Sequence verification^d^		Expressed^e^	Immunization trial
				Coverage (%)	Identity (%)		
BI-HS001	0.999 (32)	1.5791 (16)	Yes	59	100	Small	No
BI-HS002	0.999 (32)	1.0257 (34)	No	46	99	Small	No
BI-HS003	0.944 (4701)	0.9715 (38)	No	48	97	Small	No
BI-HS004	0.937 (5322)	0.9323 (49)	Yes	–	–	None	No
BI-HS005	0.930 (5459)	0.9223 (51)	No	–	–	None	No
BI-HS006	0.998 (1055)	0.8890 (56)	Yes	54	99	Small + large	No
BI-HS007	0.937 (5322)	0.8353 (73)	No	43	100	Small	No
BI-HS008	0.943 (4931)	0.8248 (86)	Yes	–	–	None	No
BI-HS009	0.998 (1055)	0.8149 (88)	No	61	99	Small + large	Yes
BI-HS010	0.943 (4931)	0.8107 (90)	Yes	–	–	None	No

^a^Rank in Vacceed scores of all 36,282 transcripts analyzed (refer to Additional file 4 for detailed information)

^b^Rank in VaxiJen scores of all 535 transcripts analyzed (refer to Additional file 4 for detailed information)

^c^Denotes whether antigen's amino acid sequence could be confirmed by mass spectrometry of proteins extracted from adult horn flies

^d^Verifying transcript expression in adult flies: Coverage denotes average % of transcript that was covered by our RT-PCR and sequencing primer sets designed for analysis of wild fly RNA. Identity denotes the average % of nucleotide identity between the sequenced region of overlap between the transcript sequence and the RT-PCR product from wild fly RNA (refer to Additional file 7: Figure S1 for detailed information)

^e^Denotes whether the antigen was successfully expressed in small-scale or large-scale *P. pastoris* expression and recombinant protein purification experiments conducted by Creative BioMart

### In silico characterization of selected antigens

The GO, InterPro, BLASTN, BLASTX, and CDD annotations, and B- and T-cell epitope predictions of the 10 selected candidates can be found in Additional file 5: Dataset S4. Of the 10 candidates, only BI-HS002 has a transmembrane helix, and all 10 have predicted signal peptides.

BI-HS001 annotation indicates involvement in the chitin-based cuticle development. BI-HS001 is similar to putative glycine-rich cell wall structural proteins of *Stomoxys calcitrans* (BLASTX e-value = 2.80E^−56^) and glycine-rich protein of *Drosophila melanogaster* (BLASTX e-value = 6.46E^−52^). BI-HS001 has 92 strong B-cell epitopes (BepiPred 2.0) and 247 and 219 linear and flexible B-cell epitopes according to BCPred and FBCpred, respectively. BI-HS001 has 51 T-cell epitopes according to NetMHC4.0 and IEDB, respectively (Additional file 5: Dataset S4).

According to GO annotation, BI-HS002 is part of the extracellular region of the cell and is involved in negative regulation of enzymes that catalyze the hydrolysis of peptide bonds. BI-HS002 contains 68 strong B-cell epitopes (BepiPred 2.0) and 43 and 56 linear and flexible B-cell epitopes according to BCPred and FBCpred, respectively. BI-HS002 has 30 (8 strong, 22 weak) and eight T-cell epitopes according to NetMHC4.0 and IEDB, respectively (Additional file 5: Dataset S4).

BI-HS003 has sequence similarity to *S. calcitrans* (BLASTX e-value = 9.18E^−22^) and *Musca domestica* (BLASTX e-value = 1.88E^−21^) putative potassium channel subfamily T member protein. BI-HS003 has 111 strong B-cell epitopes (BepiPred 2.0) and 69 and 97 linear and flexible B-cell epitopes according to BCPred and FBCpred, respectively. BI-HS003 has 36 (6 strong, 30 weak) and 28 T-cell epitopes according to NetMHC4.0 and IEDB, respectively (Additional file 5: Dataset S4).

BI-HS004 was annotated as a structural constituent of the cuticle (GO annotation) and has sequence similarity to the peritrophic matrix protein, mucin, of *Ceratitis capitata* (BLASTX e-value = 3.87E^−41^). BI-HS004 transcript has been previously reported in the larvae of *H. irritans* (BLASTN e-value = 4.68E^−90^). BI-HS004 contains 464 strong B-cell epitopes (BepiPred 2.0) and 607 and 519 linear and flexible B-cell epitopes according to BCPred and FBCpred, respectively. BI-HS004 has 143 (36 strong, 107 weak) and 152 T-cell epitopes according to NetMHC4.0 and IEDB, respectively (Additional file 5: Dataset S4).

BI-HS005 has sequence similarity to *S. calcitrans* keratin, type 1 (BLASTX e-value = 4.65E^−23^), and 61 strong B-cell epitopes (BepiPred 2.0) and 123 and 105 linear and flexible B-cell epitopes according to BCPred and FBCpred, respectively. BI-HS005 has 44 (10 strong, 34 weak) and 176 T-cell epitopes according to NetMHC4.0 and IEDB, respectively (Additional file 5: Dataset S4).

BI-HS006 has sequence similarity to adult cuticle protein 1 of *S. calcitrans* (BLASTX e-value = 4.32E^−31^), *M. domestica* (BLASTX e-value = 1.63E^−21^), and several *Drosophila* species. BI-HS006 contains a cuticle protein superfamily domain (CDD) that has shown to be involved in positive regulation of nuclear factor of activated T cells (NFAT) protein import into the nucleus according to gene ontology. NFAT proteins are implicated in the regulation of osmotic balance. BI-HS006 contained 18 strong B-cell epitopes according to BepiPred 2.0 predictions and 78 and 79 linear and flexible B-cell epitopes according to BCPred and FBCpred, respectively. BI-HS006 had 23 (5 strong, 18 weak) and 42 T-cell epitopes according to NetMHC4.0 and IEDB, respectively (Additional file 5: Dataset S4).

BI-HS007 is involved in regulation of development, oogenesis, and autophagy (GO annotation) and has sequence similarity to ecdysone-induced protein of *S. calcitrans* (BLASTX e-value = 1.18E^−42^), *M. domestica* (BLASTX e-value = 1.20E^−41^), and other Diptera. BI-HS007 contains 107 strong B-cell epitopes (BepiPred 2.0) and 81 and 76 linear and flexible B-cell epitopes according to BCPred and FBCpred, respectively. BI-HS007 has 54 (13 strong, 41 weak) and 55 T-cell epitopes according to NetMHC4.0 and IEDB, respectively (Additional file 5: Dataset S4).

BI-HS008 is annotated as a structural constituent of the vitellin membrane and is involved in construction of the vitellin membrane portion of a chorion-containing eggshell (GO annotation). BI-HS008 has sequence similarity to vitelline membrane of *D. melanogaster* (BLASTX e-value = 1.51E^−07^) and contains a vitelline membrane cysteine-rich domain (CDD). BI-HS008 has 81 strong B-cell epitopes (BepiPred 2.0) and 119 and 122 linear and flexible B-cell epitopes according to BCPred and FBCpred, respectively. BI-HS008 has 48 (13 strong, 35 weak) and 118 T-cell epitopes according to NetMHC4.0 and IEDB, respectively (Additional file 5: Dataset S4).

BI-HS009 has sequence similarity to peritrophin-48 (predicted) of *S. calcitrans* (BLASTX e-value = 7.60E^−178^)*, M. domestica* (BLASTX e-value = 8.04E^−148^), *D. obscura* (BLASTX e-value = 6.99E^−81^), and other *Drosophila* spp. According to GO annotation, its molecular function, biological process, and cellular component were chitin binding, chitin metabolic process, and the extracellular region, respectively. CDD annotation found BI-HS009 contains chitin-binding domain type 2 and chitin-binding peritrophin-A domains. BI-HS009 has 224 strong B-cell epitopes (BepiPred 2.0) and 196 and 217 linear and flexible B-cell epitopes according to BCPred and FBCpred, respectively. BI-HS009 has 97 (27 strong, 70 weak) and 177 T-cell epitopes according to NetMHC4.0 and IEDB, respectively (Additional file 5: Dataset S4).

According to GO annotation, BI-HS010 is part of the chorion, the outer shell of an insect egg. It has sequence similarity to *S. calcitrans* chorion protein (BLASTX e-value = 1.25E^−24^) and several *Drosophila* spp. CDD annotation revealed that BI-HS010 contains domains present in the C-terminal region of eukaryotic chorion protein 519. BI-HS010 contains 43 strong B-cell epitopes (BepiPred 2.0) and 63 and 64 linear and flexible B-cell epitopes according to BCPred and FBCpred, respectively. BI-HS010 has 42 (14 strong, 28 weak) and 135 T-cell epitopes according to NetMHC4.0 and IEDB, respectively (Additional file 5: Dataset S4).

### Confirmation of protein expression in adult horn flies

Using mass spectrometry, we sought to verify the presence of protein in adult horn flies that corresponded to all 10 candidate antigens. BI-HS004 and BI-HS006 were detected in both fed and unfed adult flies in the Reagent 2 fraction that was extracted with buffer containing 8 M urea and 40 mM Tris (Additional file 6: Dataset S5). BI-HS001 was found only in unfed flies in the Reagent 2 fraction (Additional file 6: Dataset S5). BI-HS008 was found only in the Reagent 2 fraction of fed flies (Additional file 6: Dataset S5). BI-HS010 was also found only in fed adult flies but in both the Reagent 1 fraction extracted with 40 mM Tris and the Reagent 2 fractions (Additional file 6: Dataset S5). BI-HS002, BI-HS003, BI-HS007, and BI-HS009 were not detected in any of the adult fly protein extracts. BI-HS005 was a special case because during the tryptic digest phase of the mass spectrometry analysis, BI-HS005 only generated three theoretical tryptic peptides of 3750 kDa and higher, which were too long and too acidic to be detected by the mass spectrometry protocol used in the present study (data not shown).

### Confirmation of transcript expression in adult flies

As described below, we successfully expressed six candidate ORFs in *P. pastoris*. We sought to verify if their corresponding transcripts could be detected in wild horn fly samples. We designed RT-PCR primers and sequencing primers to allow us to amplify and sequence at least 40% of the putative transcript corresponding to each of the six expressed candidates. Our objective was not to sequence the entire transcript that corresponded to each of our six candidate ORFs. Rather, we wanted to sequence enough of each candidate's ORF to allow us to be confident the ORF was present in the mRNA of an adult fly. Table 2 presents the expected coverage of the complete transcript resulting from our primer design. Our primers were expected to allow us to verify from 40 to 65% of the entire sequence of these six ORFs. The results of our sequencing showed that we detected all six ORFs, and the alignments of the expected transcript sequence with our sequencing data showed almost 100% identity in all cases (Table 2, Additional file 7: Figure S1).

### Recombinant expression of selected candidates in *Pichia pastoris*

Out of the 10 protein ORFs chosen for expression in *P. pastoris*, only six (BI-HS001, BI-HS002, BI-HS003, BI-HS006, BI-HS007, BI-HS009) were successfully expressed and purified from the yeast cultures at small scale (1 mg) by Creative BioMart (Table 2). And only BI-HS009 could be successfully scaled up to the 10-mg scale (Table 2) and moved forward to the immunization trial stage.

### Verification of purified recombinant antigen

**Fig. 3:**
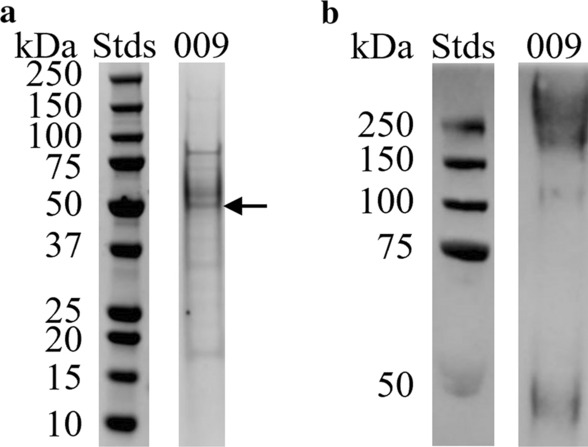
SDS-PAGE and Western blotting analysis of the BI-HS009 antigen candidate produced in *Pichia pastoris*. **a** SDS-PAGE: 4.2 µg of BI-HS009 (009) were loaded and run in NuPAGE 4–12% Bis–Tris gels, followed by staining with Coomassie Brilliant Blue R-250. Arrows indicate stained protein bands that were excised and subjected to N-terminal sequencing. **b** Western blotting detection of BI-HS009 using the Western Breeze Chromogenic Western Blot Immunodetection Kit and anti-His tag antibodies. Stds = molecular weight standards (250 kDa – 10 kDa) (Precision Plus Protein All Blue) (Bio-Rad)

Prior to the immunization trials, we sought to characterize and reverify the purified antigen solutions via PAGE, Western blotting, and N-terminal sequencing. The Coomassie-stained protein in the gel (Fig. 3a) and the anti-HisTag antibody-probed Western blotting (Fig. 3b) had a higher molecular mass than the predicted 43.58 kDa of BI-HS009. We extracted the ~ 50-kDa band for N-terminal sequencing (Fig. 3a), and the resulting 10 amino acids corresponded exactly to amino acids 23–33 of BI-HS009 (AKLNMNHICAL) (Additional file 5: Dataset S4). The first 22 amino acids of BI-HS009 were predicted to function as a signal peptide by Creative Biomart and Vacceed (Additional file 5: Dataset S4) and were probably cleaved during expression and purification of BI-HS009. Thus, the N-terminal sequencing verified the scaled-up BI-HS009 was correctly expressed and purified.

### Immunization trial

**Fig. 4:**
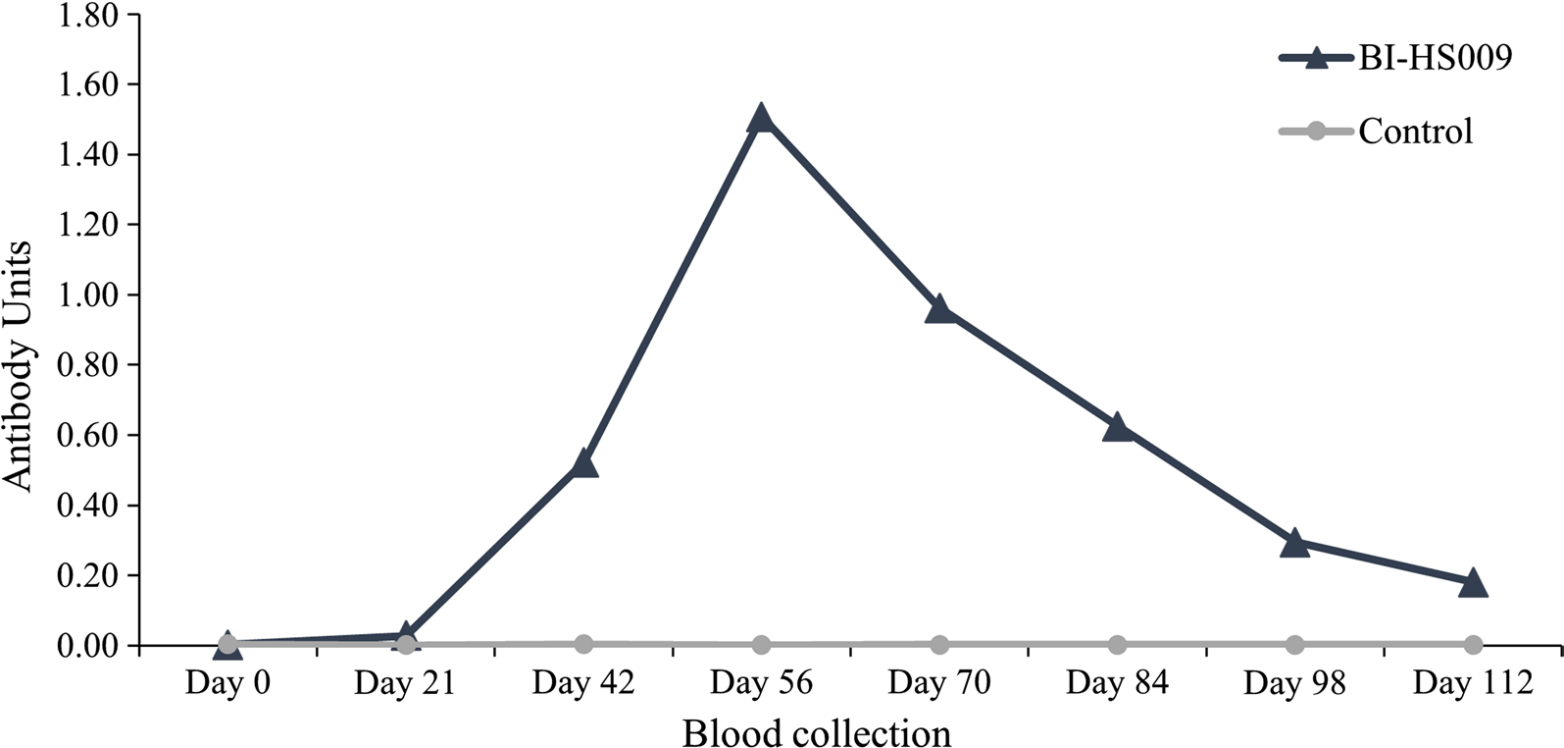
Indirect ELISA results. Animals were vaccinated with either BI-HS009 plus adjuvant (114 µg/dose, 4 ml dose) or buffer plus adjuvant only (control), and blood was collected just prior to vaccination on days 0, 21, and 42, as well as every 2 weeks after the last vaccination until day 112. Antibody titers are expressed as antibody units determined relative to a standard curve. Stars indicate significant difference (*P* < 0.05) between the control and BI-HS009 group according to two-way repeated-measures ANOVA followed by the Šídák’s multiple comparisons test. Arrows indicate vaccination days

Animals from the control group that were vaccinated with buffer and adjuvant only showed no cross-reacting antibody response to BI-HS009 in the ELISAs (Fig. 4). On the other hand, animals vaccinated with BI-HS009 had a specific IgG response, which was statistically different from the control group starting at day 42 until day 112 (ANOVA: *F*_(7, 70)_ = 18.85, *P* < 0.0001) (Fig. 4). A peak in the immune response was observed on day 56, and over 2 months after the final vaccination (day 112), a statistically significant immune response was still evident in the vaccinated cattle (Fig. 4).

### Protective efficacy against horn fly

According to the multiple logistic regression analysis, blood from animals vaccinated with BI-HS009 negatively affected development of egg to pupariation (odds ratio: 0.7215, 95% confidence interval = 0.5296–0.9855, *P* = 0.0392) as well as puparia to adult (odds ratio: 0.5360, 95% confidence interval = 0.3925–0.7322, *P* < 0.0001) independently of day of blood collection (odds ratio: 1.005, 95% confidence interval = 0.9944–1.016, *P* = 0.3521 and odds ratio: 0.9937, 95% confidence interval = 0.9829–1.005, *P* = 0.2562 for egg to pupariation and puparia to adult development, respectively).

At day 56, flies feeding upon blood from cattle vaccinated with BI-HS009 produced 37 and 43% of the pupae and newly emerged adults, respectively, produced by flies feeding upon blood from cattle vaccinated with adjuvant only, a significant difference (ANOVA: *F*_(1, 6)_ = 8.221, *P* = 0.028 and *F*_(1, 6)_ = 8.299, *P* = 0.028) (Table 3). No difference was observed for those parameters at days 21 and 42 (Table 3).

**Table 3:** Results of in vitro feeding assay with adult flies fed with blood from vaccinated animals (BI-HS009 or control) collected on days 21, 42, and 56

	BI-HS009^a^		Control^a^		ANOVA
	Total	Mean (SD)	Total	Mean (SD)	
Fly mortality					
Day 21	6	1.50 (1.29)	1	0.25 (0.50)	*F*_(1, 6)_ = 2.000, *P* = 0.207
Day 42	4	1.00 (0.82)	5	1.25 (0.96)	
Day 56	5	1.25 (0.96)	3	0.75 (0.96)	
Number of eggs laid					
Day 21	133	33.3 (22.2)	259	64.8 (20.6)	*F*_(1, 6)_ = 4.447, *P* = 0.079
Day 42	195	48.8 (21.4)	189	47.3 (27.2)	
Day 56	114	28.5 (18.5)	303	75.8 (26.7)	
Number of pupae					
Day 21	106	26.5 (17.7)	217	54.3 (16.1)	*F*_(1, 6)_ = 8.221, *P* = 0.028
Day 42	155	38.8 (16.2)	163	40.8 (23.7)	
Day 56	95	23.8 (13.3)^*^	260	65.0 (16.7)	
Number of newly emerged adults					
Day 21	83	20.8 (15.6)	198	49.5 (13.5)	*F*_(1, 6)_ = 8.299, *P* = 0.028
Day 42	83	20.8 (15.0)	119	29.8 (24.0)	
Day 56	92	23.0 (13.5)^*^	214	53.5 (15.3)	

*SD* standard deviation

*Indicates significant difference at *P* < 0.05 between BI-HS009 and control group according to two-way repeated-measures ANOVA followed by the Šídák’s multiple comparisons test

^a^Four cages with 20 flies each (10 males and 10 females) were used per group (BI-HS009 and control) for each blood collection date (days 21, 42, or 56)

## Discussion

After a thorough evaluation of more than 79,000 transcript sequences using a reverse vaccinology approach comprising three vaccine prediction and 11 annotation tools (Fig. 1; Table 1), we identified an anti-horn fly antigen candidate that caused a significant antibody response in vaccinated animals (Fig. 4) and significantly reduced the number of pupae and adults that developed from adults fed on blood from vaccinated animals when compared to a control group in an in vitro feeding assay (Table 3).

Although we had 100 sequences that met Vaxign, Vacceed, and VaxiJen selection criteria (Fig. 1, Additional file 4: Dataset S3), we could evaluate only 2–4 antigens in immunization trials. Our experience had been that candidate ORFs could be successfully expressed in *P. pastoris* with sufficient yields for efficacy trials and purified via His-tag technology only ~ 50% of the time. Thus, after careful evaluation of the annotation of each of those 100 ORFs (Additional file 4: Dataset S3), we manually picked 10 ORFs as our vaccine candidates and tried to express them in *P. pastoris.* Only one of them, BI-HS009, could be expressed at enough quantities for the immunization trial (Fig. 1; Table 2, Additional file 5: Dataset S4).

BI-HS009 was chosen as one of the top 10 candidates due to its lack of transmembrane domains and presence of signal peptides, as well as similarity to peritrophin-48 of several dipterans and presence of chitin-binding type 2 and peritrophin-A domains (Additional file 4: Table S2). Further annotation revealed that BI-HS009 might be involved in the chitin metabolic process and enable chitin binding (Additional file 5: Dataset S4). This candidate had the first and second highest number of T-cell and strong B-cell epitopes according to BepiPred 2.0 and IEDB-MHCI, respectively (Additional File 5: Dataset S4). Although, BI-HS009 was not found in proteins extracted from adult flies according to the mass spectrometry analysis (Additional file 6: Dataset S5), we were able to confirm BI-HS009 transcript expression in adult flies using RT-PCR and DNA sequencing (Additional file 7: Fig. S1). It is possible that the protein purification method we used or the trypsin digestion used for preparing the samples for mass spectrometry were not suitable for the BI-HS009 protein. Peritrophins are highly glycosylated [59, 60], and BI-HS009 seems to follow the same rule as shown by the higher molecular weight in the SDS-PAGE and Western blotting (Fig. 3) compared with its predicted weight. The glycan groups present in the protein may have hampered the digestion process with trypsin affecting its identification by mass spectrometry [61].

Functional studies in the sheep blow fly, *Lucilia cuprina* (Diptera: Calliphoridae), and the old world screw worm, *Chrysomya bezziana* (Diptera: Calliphoridae), have shown that peritrophin-48 is an integral protein of the peritrophic matrix and is likely related to the maintenance of the peritrophic matrix’s shape, strength, elasticity, and porosity [59, 60]. The peritrophic matrix is a noncellular semipermeable layer composed of chitin and glycoproteins that line the midgut of most invertebrates, that is critical to insects’ digestive process and protection against microorganisms, parasites, and toxins [62].

Due to its importance to the peritrophic matrix, peritrophins have been investigated as vaccine candidates. Casu et al. [63] showed that growth of *L. cuprina* first-instar larvae was inhibited by anti-peritrophin-95 antibodies in a dose-dependent manner in an in vitro assay: serum from sheep vaccinated with peritrophin-95 caused 60% reduction in larval weight, and serum enriched by two- or fourfold of anti-peritrophin-95 antibodies reduced larval weight by 86 and 98%, respectively. The authors attributed the results to an antibody-mediated blockage of the normally semipermeable peritrophic matrix limiting the availability of nutrients to the larvae [63]. In the old-world screw worm, *C. bezziana*, vaccination with the whole native peritrophic membrane affected larval weight in vitro and in vivo, but this deleterious effect was not observed when recombinant peritrophins were used [64, 65]. Vaccination of cattle with a crude extract of peritrophins (65–75 kDa) isolated from adult *Haematobia irritans exigua* did not affect fly mortality or fecundity (total number of pupae, mean weight of pupae, and percentage emergence of pupae) in either in vitro or in vivo assays, despite good antibody responses in the vaccinated animals [66].

In the present study, feeding flies with blood from animals vaccinated with recombinant BI-HS009 collected on the day of highest antibody response (day 56, Fig. 4) significantly reduced the number of progeny pupae and emerging adults by 63 and 57%, respectively (Table 3). In addition, multiple logistic regression analysis showed that the relative odds ratio of pupae (odds ratio: 0.7215, 95% confidence interval = 0.5296–0.9855, *P* = 0.0392) and adult (odds ratio: 0.5360, 95% confidence interval = 0.3925–0.7322, *P* < 0.0001) development was significantly smaller for flies fed on blood from animals vaccinated with recombinant BI-HS009 compared to flies that fed on blood from animals vaccinated with adjuvant only. However, pupae and the emerging adults did not have direct contact with the anti-BI-HS009 antibodies, an interesting finding that deserves further evaluation.

To the best of our knowledge, attempts to develop an anti-horn fly vaccine have been restricted to studies with crude antigens extracted from the horn fly’s gut, recombinant thrombostasin (an anti-clotting protein found in horn fly saliva), and hematobin (a salivary gland protein) [13–16]. These studies have shown that vaccination of cattle with crude or recombinant antigens induces a specific and significant IgG response, similar to what we found in the present study (Fig. 4). However, only Breijo et al. [16] showed a direct effect on fly infestation as a result of vaccination, while the other studies reported only a reduced number of eggs [13] or reduced blood intake and delayed ovarian development [14, 15], but no direct negative effects on fly mortality, as also observed in the present study (Table 3).

The present study was the first to measure the effects of vaccination on different life stages of the horn fly (adults, eggs, and pupae) (Table 3) and to show that a significant IgG response was still observed in the BI-HS009-vaccinated animals more than 2 months after the third vaccination when compared with the control group (Fig. 4). However, by collecting eggs only on days 6–8 in the in vitro feeding assay, we were not able to evaluate whether feeding upon the BI-HS009-vaccinated bovine blood (compared to feeding upon adjuvant only-vaccinated blood) merely delayed oviposition, without an actual decrease in total egg yield. This would also have impacted the findings on pupal production and emerging adult totals from each cage and must be further investigated.

Very few anti-arthropod vaccines have been developed, and only a single vaccine has successfully and sustainably reached the global market. Allen and Humphreys [67] immunized mammalian hosts with protein extracts from partially fed ticks and discovered that ticks feeding upon the vaccinated hosts showed significantly reduced reproductive performance. Willadsen et al. [68] discovered the tick protein Bm86 was an effective antigen as a component of an anti-tick vaccine, leading to the anti-tick vaccine TickGARD [69], soon followed by the anti-tick vaccine Gavac™ [70]. An effective vaccine against the cattle grub, *Hypoderma lineatum*, was developed in the 1980s, but market factors prevented commercialization [71].

Those vaccines were initially discovered by fractionating extracts of biological material, eventually testing purified protein from these fractions for anti-arthropod efficacy, a lengthy process. The reverse vaccinology approach provided a means to shorten the vaccine antigen discovery phase and take advantage of the horn fly genome and transcriptome datasets generated in our laboratory [17, 19]. We found that while most of the computational aspects of this research could proceed quickly, some significant problems still exist that need resolution or workarounds to facilitate anti-arthropod vaccine research. The primary bottleneck we experienced was during the recombinant protein expression phases. Besides their predicted biological functions, one of the main characteristics we used for the selection of the 10 candidates were the presence of signal peptides and the lack of transmembrane helices, because they are known to affect in vitro expression of proteins [24, 25]. Even though all the selected candidates had predicted signal peptides, and none had transmembrane helices, except for BI-HS002 (Additional file 5: Dataset S4), only one, BI-HS009, was successfully scaled up to enable evaluation of efficacy in vaccinated bovines (Table 2). Since the anti-tick vaccine Gavac™ is produced in *P. pastoris* [70] and this yeast is reportedly involved in the glycosylation of proteins, which can improve immunogenicity, we decided *P. pastoris* would be our expression system of choice. But considering the promising features of the other candidates (Table 2, Additional file 5), we believe different expression systems such as *Escherichia coli* or insect cells should be tried, or even a change in strategy by trying to express a recombinant protein constructed using only the most immunogenic epitopes of each of the selected candidates could be pursued.

Despite being a valuable alternative for parasite control, vaccines do not offer the same knockdown effects as insecticides, but rather affect the parasite population over time [7, 8]. The BI-HS009 vaccine caused a 63 and 57% reduction in the progeny pupae and newly emerged adults (Table 3), respectively, which would be the next fly generation. Thus, although immediate fly mortality might not be achieved by available vaccine antigens, such as thrombostasin [14, 15], hematobin [16] or peritrophins, fly populations might be reduced below economic thresholds over time, providing cattle ranchers with another option for fly control.

## Conclusions

The reverse vaccinology approach helped narrow the search for anti-horn fly vaccine candidates from more than 79,000 transcript sequences to our selected top 100 candidates. Further annotation was necessary to better characterize each candidate and choose a feasible number for in vitro recombinant protein expression, a crucial step that limited the number of antigens that could reasonably be tested in the immunization trials. Although vaccination with BI-HS009, a putative peritrophin, did not affect fly mortality in in vitro assays, it elicited a significant antibody response in vaccinated animals and significantly affected pupal survival and adult emergence, demonstrating the potential of this antigen as a vaccine candidate.

## Supplementary Information

**Additional file 1**: **Dataset S1**. Horn fly contigs used in the in silico analysis.

**Additional file 2**: **Dataset S2**. Horn fly longest ORFs translated with Virtual Ribosome.

**Additional file 3**: **Table S1**. Primers used for RT-PCR and sequencing of adult *Haematobia irritans*.

**Additional file 4**: **Dataset S3**. Vaxign, Vacceed, and Vaxign results for all ORFs analyzed with these tools, and Gene Ontology, InterPro, BLASTN and BLASTX annotations for the 100 ORFs with highest VaxiJen score.

**Additional file 5**: **Dataset S4**. Transcript and ORF sequences of the 10 vaccine candidate antigens selected for expression in Pichia pastoris, including their respective BLASTX, BLASTN, InterPro, GO and CDD annotations, and B cell and T cell epitope predictions.

**Additional file 6**: **Dataset S5**. Results of the mass spectrometry analysis. The LC/MS data were searched against multiple custom databases, including the NCBI protein database (4837 horn fly sequences as of January 08, 2018) and the databases used in the in silico analysis.

**Additional file 7**: **Figure S1**. Nucleotide sequencing verification alignments for the six *Haematobia irritans* antigens successfully expressed in *Pichia pastoris*. Primers used for PCR and sequencing are shown for each transcript. Refer to Additional file 3: Table S1 for details about the primers. In the aligned sequences, the sequence of the vaccine candidate antigen is in the top row and underneath are the various sequences resulting from RT-PCR whereby we sought to verify the presence of the antigen's ORF-encoding transcript in various wild horn fly populations: Saint Gabriel (females), Rosepine, and Super Resistant.

## Data Availability

All data generated or analyzed during this study are included in this published article and its supplementary information files.

## Abbreviations

ORFs: Open reading frames
RT-PCR: Reverse transcription polymerase chain reaction
PCR: Polymerase chain reaction
RNA: Ribonucleic acid
IgG: Immunoglobulin G
DDT: Dichlorodiphenyltrichloroethane
TSA: Transcriptome Shotgun Assembly Sequence Database
MHC: Major histocompatibility complex
BLAST: Basic Local Alignment Search Tool
BLASTN: Nucleotide Basic Local Alignment Search Tool
BLASTX: Translated Basic Local Alignment Search Tool
GO: Gene Ontology
mRNA: Messenger ribonucleic acid
EST: Expressed sequence tags
EMBL: European Molecular Biology Laboratory
DDBJ: DNA Data Bank of Japan
NCBI: National Center for Biotechnology Information
NR: Non-redundant
CDS: Coding sequence
PDB: Protein Data Bank
Swiss-Prot: Knowledge base containing manually curated protein sequences
PIR: Protein Information Resource
PRF: Protein Research Foundation
WGS: Whole genome sequencing
CDD: Conserved Domain Database
Pfam: Protein families
SMART: Simple Modular Architecture Research Tool
COG: Clusters of Orthologous Groups of proteins
PRK: Protein k(c)lusters
TIGRFAMS: The Institute for Genomic Research's database of protein families
PSSMS: Position-specific scoring matrix
IEDB: Immune Epitope Database
USDA: United States Department of Agriculture
ARS: Agricultural Research Service
KBUSLIRL: Knipling-Bushland United States Livestock Insects Research Laboratory
SDS: Sodium dodecyl sulfate
SDS-PAGE: Sodium dodecyl sulphate–polyacrylamide gel electrophoresis
MS: Mass spectrometry
LC/MS: Liquid chromatography–mass spectrometry
DNA: Deoxyribonucleic acid
cDNA: Complementary DNA
LSU: Louisiana State University
dNTPs: Deoxyribonucleotide triphosphate
BMGY: Buffered glycerol complex medium
BMMY: Buffered methanol complex medium
Ni^2^^+^-NTA resin: Nickel-charged affinity resin (NTA-nitrilotriacetic acid)
PBS: Phosphate-buffered saline
CAPS: *N*-Cyclohexyl-3-aminopropanesulfonic acid
TS6: Boehringer Ingelheim’s proprietary adjuvant
ELISA: Enzyme-linked immunosorbent assay
TBS: Tris-buffered saline
TMB: 3,3′,5,5′-Tetramethylbenzidine
OD450: Optical density 450 nm
contig ID: Contig identification
SD: Standard deviation
