# Supplementary material for: Identification of anti-horn fly vaccine antigen candidates using a reverse vaccinology approach
Source: Parasit Vectors. 2021 Sep 3;14:442. doi: 10.1186/s13071-021-04938-5 (PMC8414034; doi:10.1186/s13071-021-04938-5)
Supplement: Supplementary file 3 — Additional file 3: Table S1. Primers used for RT-PCR and sequencing of adult Haematobia irritans. [file 13071_2021_4938_MOESM3_ESM.docx]

| **Antigen**  **(length)** | **Primer ID** | **Primer Sequence** | **Annealing Temperature (°C)** | **Number of PCR cycles** | **Amplicon length (bp)** |
| --- | --- | --- | --- | --- | --- |
| BI-HS001 | LD29 | 5’-ACCTGGAGGCAGTGGT-3’ | 56 | 35 | 756 |
| (843 bp) | LD30 | 5’-GATTTTCCGGGAGGAC-3’ |  |  |  |
|  | LD31 | 5’-GGTAGCTCCGGTGGA-3’ | 62 | 35 | 518 |
|  | LD32 | 5’-GCAGCTGTGGGTATGAC-3’ |  |  |  |
|  | BIHS001F | 5’-GATTTGGCGGCAGTTC-3’ | Used for sequencing | | 400 |
|  | BIHS001R | 5’-GGAGCCTGGTAAGATGGAG-3’ |  |  |  |
| BI-HS002 | LD33 | 5’-CAACAATTGGCCTTC-3’ | 55 | 35 | 527 |
| (660 bp) | LD34 | 5’-AGGTGATCAGTTGGG-3’ |  |  |  |
|  | LD35 | 5’-CAAGATCACCGGTTC-3’ | 56 | 35 | 408 |
|  | LD36 | 5’-GCGCTTACTGAAAATG-3’ |  |  |  |
|  | BIHS002F | 5’-GGATCCAATACCTAGACC-3’ | Used for sequencing | | 353 |
|  | BIHS002R | 5’-CTTGCATTATTCGCC-3’ |  |  |  |
| BI-HS003 | LD37 | 5’-GATAATCCTACGTCTGTG-3’ | 56 | 30 | 448 |
| (563 bp) | LD38 | 5’-TCATCATCACTTTCGTC-3’ |  |  |  |
|  | LD39 | 5’-ATGCAACGCAAACACC-3’ | 63 | 30 | 370 |
|  | LD40 | 5’-TCGCCATCATCATTACC-3’ |  |  |  |
|  | LD69 | 5’-GCAACGACTACAACCACT-3’ | 63 | 30 | 327 |
|  | LD70 | 5’-ACTAAACGAGGAGGAGG-3’ |  |  |  |
|  | LD84 | 5’-CAACCACCATACCTCTG-3’ | Used for sequencing | | 274 |
|  | LD85 | 5’-ACTGGGAGTATTGAAGGTC-3’ |  |  |  |
| BI-HS006 | LD49 | 5’-AAGAGTGAAAGATGCAGG-3’ | 59 | 35 | 530 |
| (665 bp) | LD50 | 5’-CGCTTTTGCCGTAGTC-3’ |  |  |  |
|  | LD51 | 5’-GAGAAGAGTGTCGGAGTATG-3’ | 62 | 35 | 489 |
|  | LD52 | 5’-GTTGGCCCTTGCTGT-3’ |  |  |  |
|  | BIHS006F | 5’-GATTGTTGTTGCAGAAATC-3’ | Used for sequencing | | 390 |
|  | BIHS006R | 5’-AGCTCATGGTGCTTGG-3’ |  |  |  |
| BI-HS007 | LD53 | 5’-TCACCCACACAACATC-3’ | 56 | 35 | 452 |
| (606 bp) | LD54 | 5’-TGATCCCGATCAAATG-3’ |  |  |  |
|  | LD55 | 5’-AACAACAACAGCACCAG-3’ | 59 | 35 | 326 |
|  | LD56 | 5’-TGATGGATGTGTCGAAC-3’ |  |  |  |
|  | LD77 | 5’-CAACAACAACAGCACC-3’ | 54 | 35 | 403 |
|  | LD78 | 5’-TTCCACGATTCCAAA-3’ |  |  |  |
|  | LD79 | 5’-GCTTGGTGGAGGTGG-3’ | Used for sequencing | | 342 |
|  | LD80 | 5’-TTCGGATGCAGAAATG-3’ |  |  |  |
| BI-HS009 | LD61 | 5’-CATGTGCCTTGATTTTG-3’ | 56 | 35 | 1217 |
| (1431 bp) | LD62 | 5’-AGCATCTGTTGTGGCT-3’ |  |  |  |
|  | LD63 | 5’-GGTCTAATGATTTCCTCTG-3’ | 57 | 35 | 1038 |
|  | LD64 | 5’-GCCATCGGTTGTTTC-3’ |  |  |  |
|  | BIHS009F | 5’-TGCTCCAGGCTATTTC-3’ | Used for sequencing | | 860 |
|  | BIHS009R | 5’-CACCCCTGACACAAG-3’ |  |  |  |
