# Supplementary material for: Identification of anti-horn fly vaccine antigen candidates using a reverse vaccinology approach
Source: Parasit Vectors. 2021 Sep 3;14:442. doi: 10.1186/s13071-021-04938-5 (PMC8414034; doi:10.1186/s13071-021-04938-5)

BI-HS001 Nucleotide Alignments

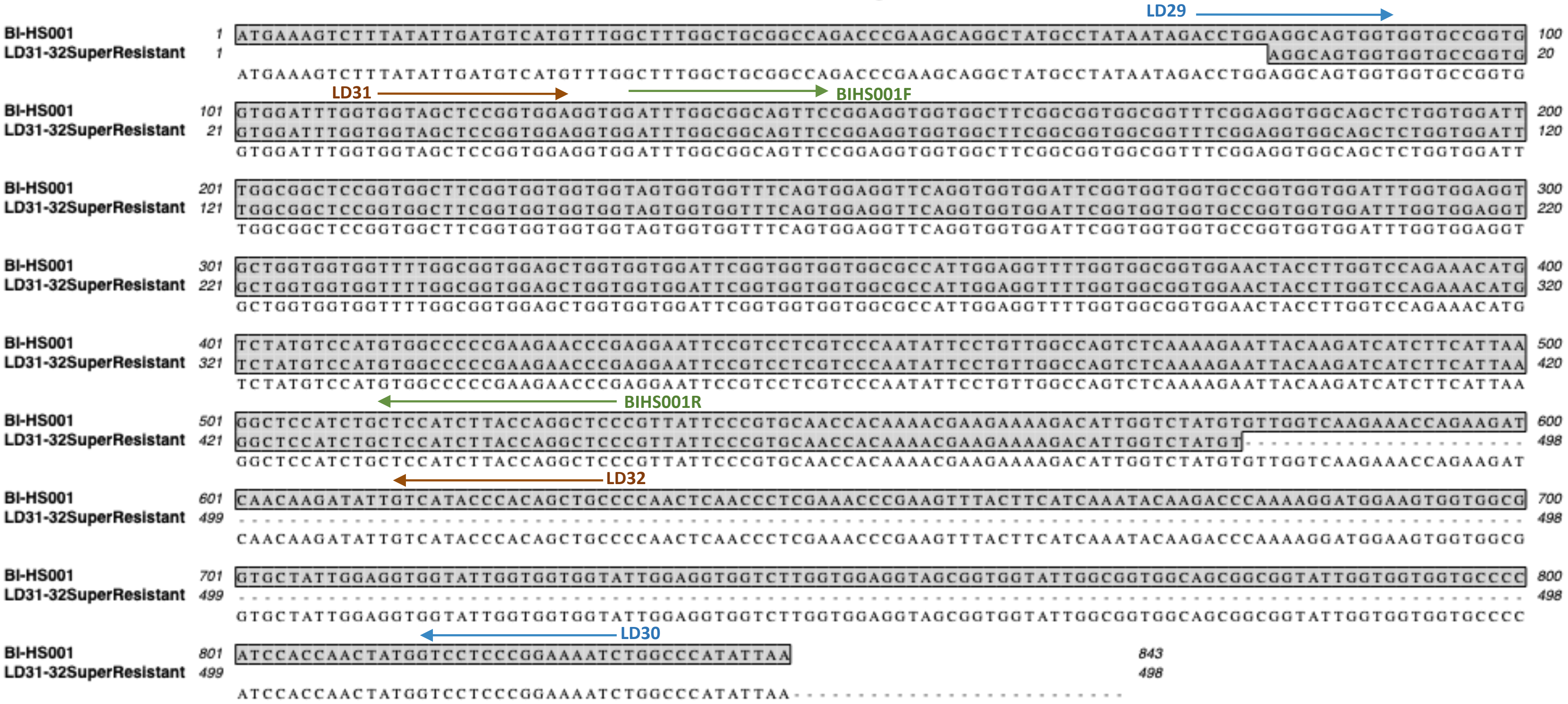

BI-HS002 Nucleotide Alignments

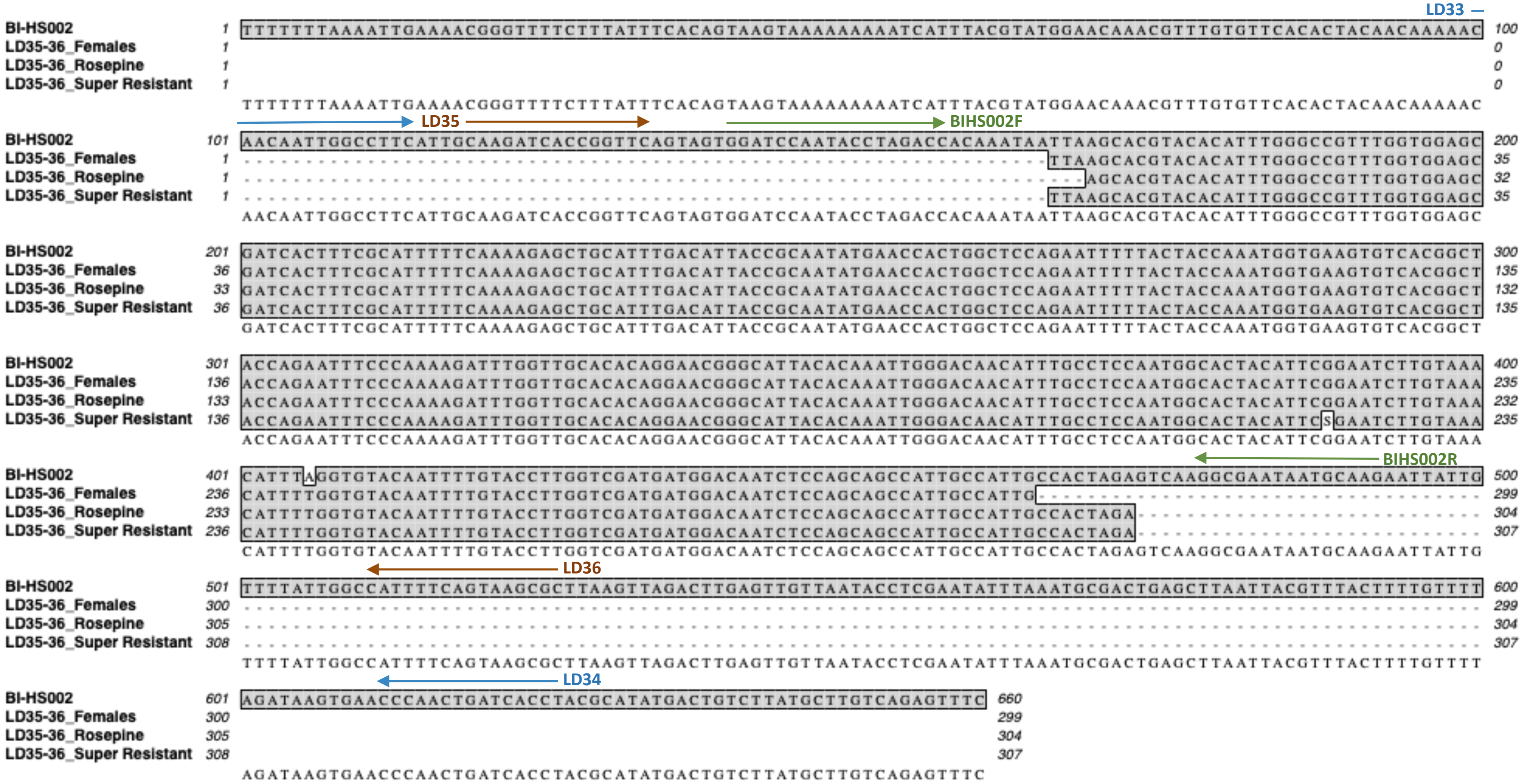

BI-HS003 Nucleotide Alignments

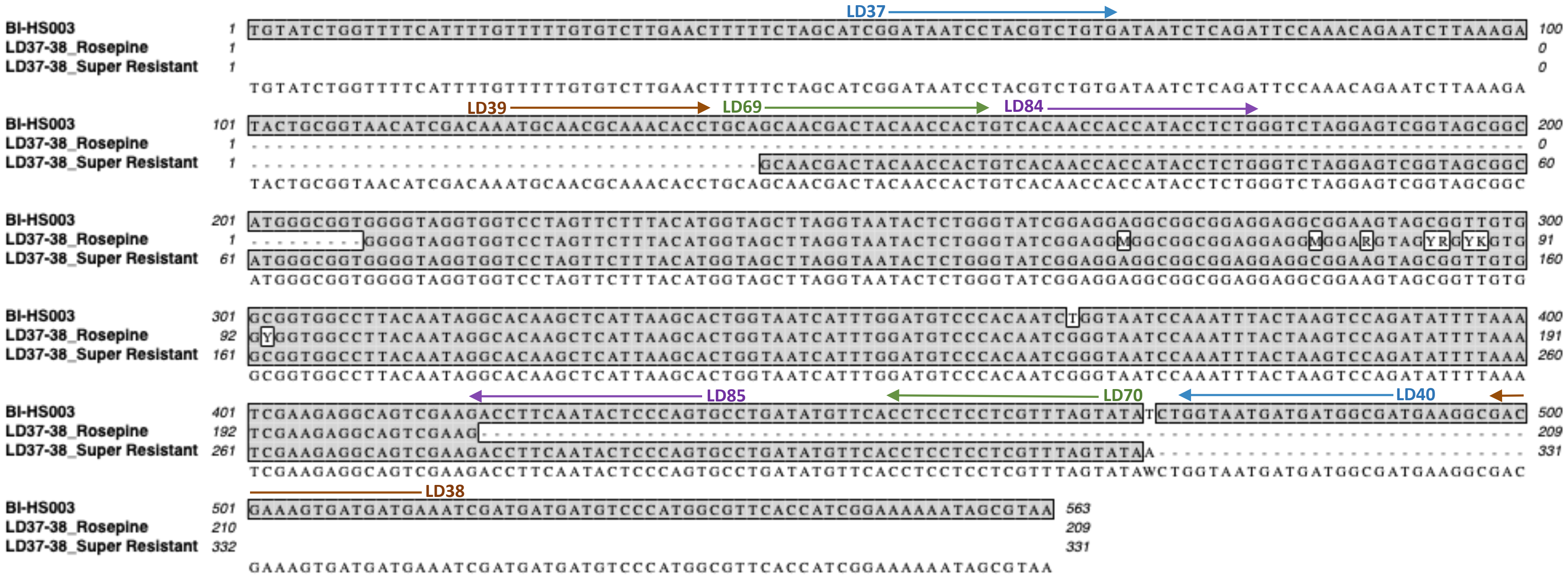

BI-HS006 Nucleotide Alignments

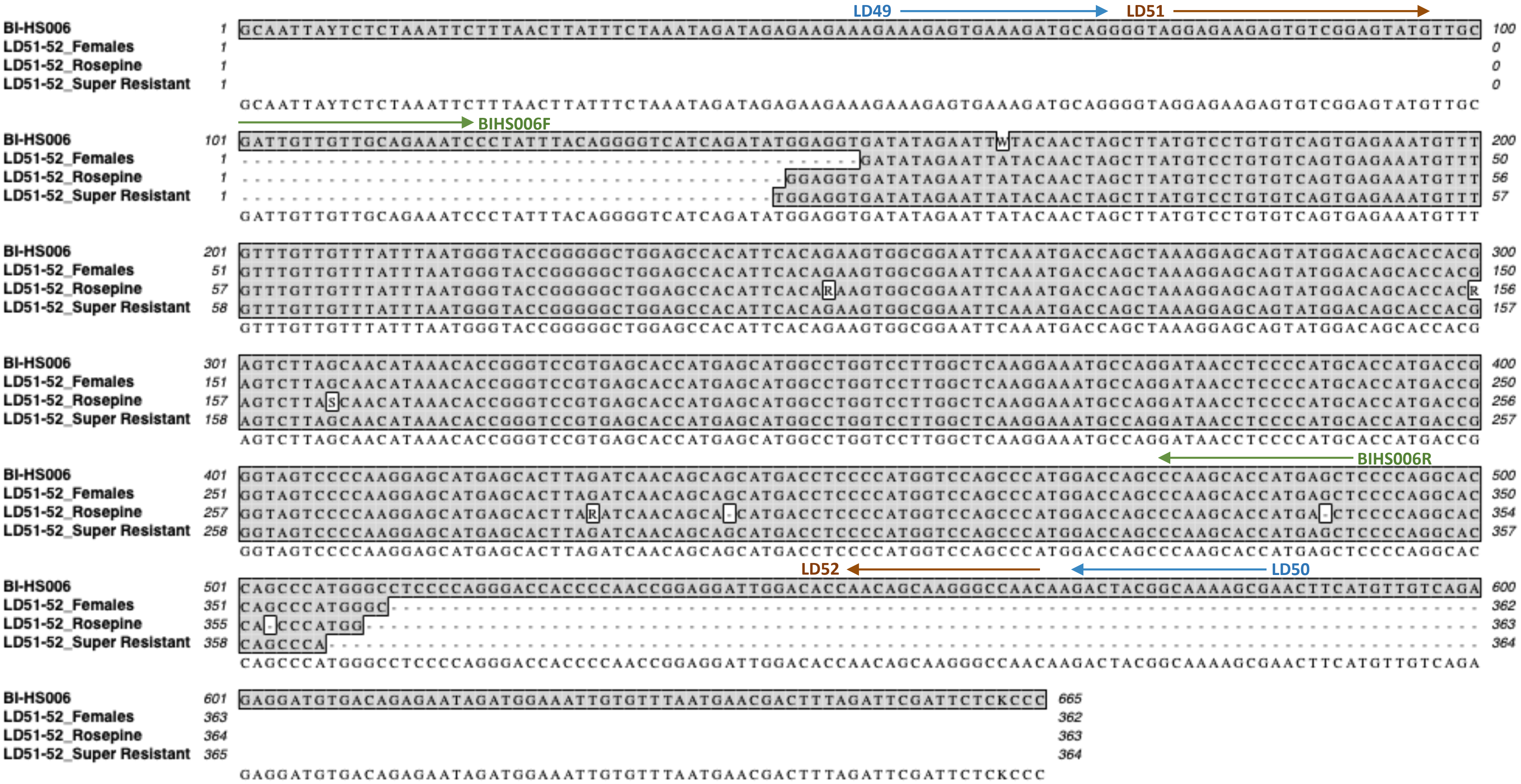

BI-HS007 Nucleotide Alignments

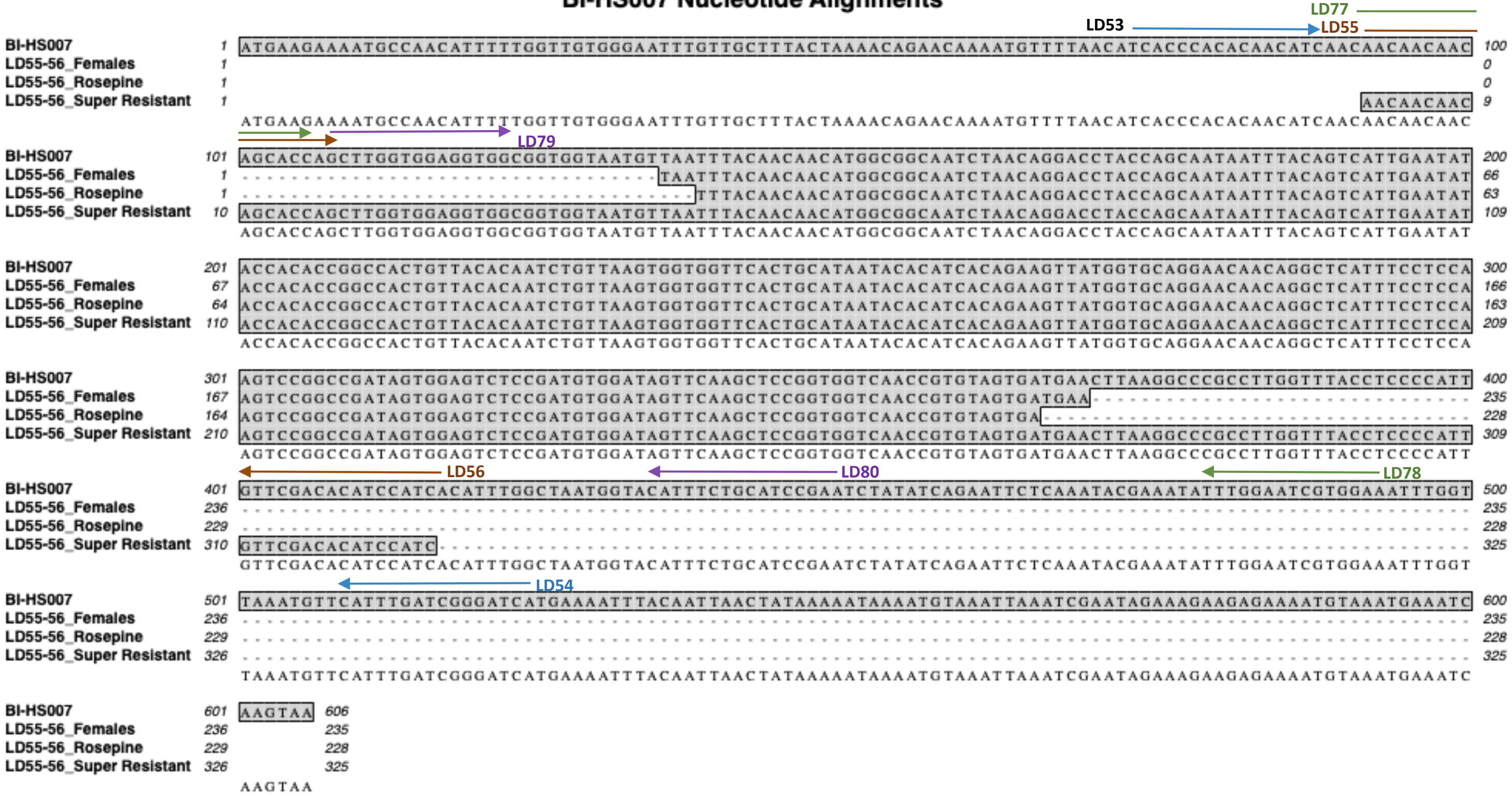

BI-HS009 Nucleotide Alignments

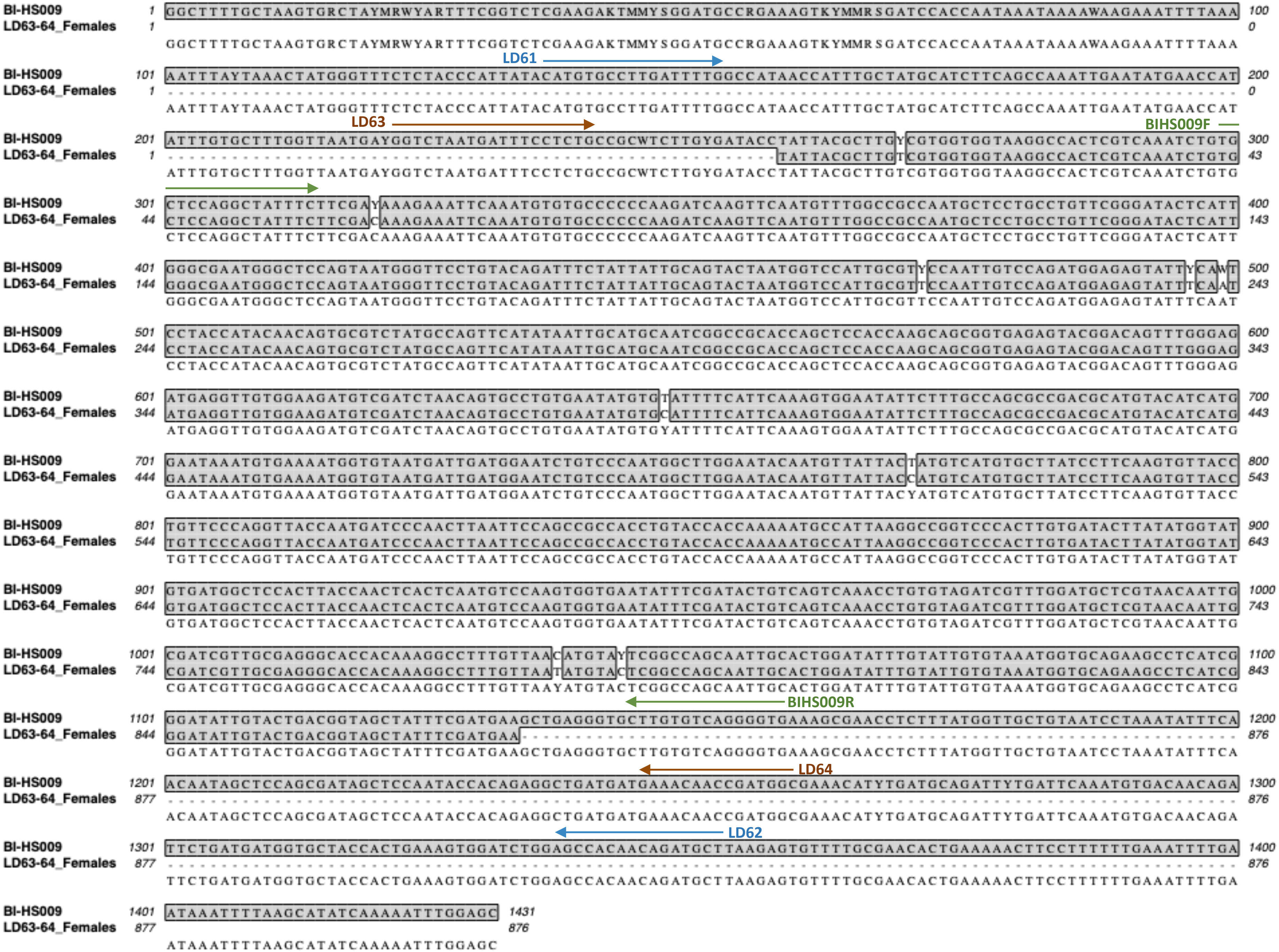

Supplement: Supplementary file 7 — Additional file 7: Figure S1. Nucleotide sequencing verification alignments for the six Haematobia irritans antigens successfully expressed in Pichia pastoris. Primers used for PCR and sequencing are shown for each transcript. Refer to Additional file 3: Table S1 for details about the primers. In the aligned sequences, the sequence of the vaccine candidate antigen is in the top row and underneath are the various sequences resulting from RT-PCR whereby we sought to verify the presence of the antigen's ORF-encoding transcript in various wild horn fly populations: Saint Gabriel (females), Rosepine, and Super Resistant. [file 13071_2021_4938_MOESM7_ESM.pdf]
